# Enhancing economic competitiveness analysis through machine learning: Exploring complex urban features

**DOI:** 10.1371/journal.pone.0293303

**Published:** 2023-11-07

**Authors:** Xiaofeng Xu, Zhaoyuan Chen, Shixiang Chen

**Affiliations:** 1 School of Political Science and Public Administration, Wuhan University, Wuhan, Hubei, China; 2 Development Research Center, Qingdao City Construction Investment (Group) Co., Ltd., Qingdao, Shandong, China; University of California Los Angeles, UNITED STATES

## Abstract

Urban economic competitiveness is a fundamental indicator for assessing the level of urban development and serves as an effective approach for understanding regional disparities. Traditional economic competitiveness research that relies solely on traditional regression models and assumes feature relationship theory tends to fall short in fully exploring the intricate interrelationships and nonlinear associations among features. As a result, the study of urban economic disparities remains limited to a narrow range of urban features, which is insufficient for comprehending cities as complex systems. The ability of deep learning neural networks to automatically construct models of nonlinear relationships among complex features provides a new approach to research in this issue. In this study, a complex urban feature dataset comprising 1008 features was constructed based on statistical data from 283 prefecture-level cities in China. Employing a machine learning approach based on convolutional neural network (CNN), a novel analytical model is constructed to capture the interrelationships among urban features, which is applied to achieve accurate classification of urban economic competitiveness. In addition, considering the limited number of samples in the dataset owing to the fixed number of cities, this study developed a data augmentation approach based on deep convolutional generative adversarial network (DCGAN) to further enhance the accuracy and generalization ability of the model. The performance of the CNN classification model was effectively improved by adding the generated samples to the original sample dataset. This study provides a precise and stable analytical model for investigating disparities in regional development. In the meantime, it offers a feasible solution to the limited sample size issue in the application of deep learning in urban research.

## Introduction

Scholars have long argued that competition between local governments in China over economic development is one of the keys to understanding China’s economic system. Fierce economic competition among Chinese cities has led to the promotion of economic competitiveness as a starting point for most local government policies [1]. In such a situation, a more efficient and accurate method of distinguishing differences in urban economic competitiveness is essential, whether it is government policymaking and performance evaluation or industrial capital and population movement decisions.

The existing research on urban economic competitiveness can be categorized into two directions. One of which is to explore the factors affecting competitiveness and their intrinsic relationships, including human capital, education system, and infrastructure, which have been proven to effectively influence urban economic competitiveness [2, 3]. These causal relationships are typically validated through traditional mathematical models such as regression models. Another research direction is to construct a system of quantifiable indicators to assess and rank the competitiveness of economies at the same level within a region [4]. This type of research analyzes and selects the influencing factors using an explanatory framework, and calculates the weighting parameters by applying variance weighting, entropy weighting, and principal component scoring methods.

These methods assume a linear relationship between the dependent variable and the independent variables. While the dependent variable is influenced by a single independent variable, it is also affected by the interaction effects among multiple independent variables. Therefore, traditional approaches require the artificial determination of the relationship between urban economy and urban characteristics, as well as the interaction effects among urban characteristics, before analyzing urban economic competitiveness. However, cities are complex systems composed of factors such as infrastructure, environment, and population [5]. Without a complete understanding of how various urban characteristics interact within this system, it is extremely challenging to specify the interrelationships among factors in the model. This limitation restricts traditional methods to examining only a limited set of influencing factors for economic competitiveness and prevents the capture of complex nonlinear relationships and interaction effects among these factors.

The development of deep learning neural networks has provided a new approach to address this dilemma. By considering complex urban features as input variables and using indicators such as urban economic level and variations in urban development as output labels, it becomes possible to incorporate urban features into the processing architecture of neural networks [6]. On the other hand, neural networks can automatically perform nonlinear modeling of complex interaction patterns among input features [7]. This automated learning approach enables efficient and accurate analysis of problems based on intricate urban features, without the need for assumptions regarding the interrelationships of urban features based on existing theories [8].

A neural network is a computational model that simulates the structure of a biological nervous system. It has developed over the years, with the invention of backpropagation algorithms, regularization techniques, varieties of activation functions, and improvements in computer performance, neural networks acquired the potential for large-scale data applications. Recently, the rise of deep learning neural networks with more hidden layers and increasingly complex model architectures has led to revolutionary advancements in computer vision and natural language processing, making neural networks the core technology driving the development of artificial intelligence. Among the numerous models derived from deep neural networks, CNN has emerged as one of the most influential innovative technologies in the field of deep learning, owing to its powerful feature extraction capability. The basic principle of a CNN is to use a multilayer convolutional structure to extract data features and compose a high-dimensional feature map for recognition and classification. The application of convolution and pooling computations effectively reduces the number of model weight parameters and improves the anti-interference capability [9]. This multilayer convolutional structure allows for the identification of the most prominent features within the data, providing CNN with exceptional feature extraction capabilities and computational efficiency, which has resulted in the widespread application of CNN in the field of feature engineering [10]. The utilization of CNN for feature extraction has demonstrated remarkable performance and extensive applicability in various domains, including urban pollution prediction [11], traffic flow analysis [12], land use classification [13], and others. Hence, it is plausible to believe that in economic research based on complex urban features, CNN possess the capability to extract the core features from data and achieve precise classification of economic competitiveness. This ability has also been a key consideration in selecting the model for this study.

The key to machine learning methods lies in modeling and extracting features from the data to achieve effective model construction and data learning. As a consequence, the size of the sample becomes a fundamental factor influencing the performance of machine learning models like CNN [14]. However, in the case of a specific region, the number of cities remains fixed. Even by combining data from different years, the amount of data available is still limited. In such circumstances, traditional solutions predominantly focus on optimizing model architecture and training methods. However, the generalization ability and practical effectiveness of these methods are not particularly remarkable. The emergence of generative adversarial network (GAN) provides another solution to this problem, that is expanding the training sample size by generating realistic data. GAN, which was invented by Goodfellow et al. [15], is a generative modeling framework consisting of a generator and discriminator in competing states. The generator is responsible for generating fake data and tricking the discriminator, whereas the objective of the discriminator is to accurately distinguish between real and fake data. The generator progressively enhances its performance through competition with the discriminator. Upon completion of training, the discriminator becomes capable of producing pseudo-realistic data that closely resembles the original data, while also incorporating additional data features. By utilizing these generated data samples to expand the sample size of the original data, the performance of the CNN model is further improved. Shorten and Khoshgoftaar [16] compared a variety of approaches to improve model performance, where GAN demonstrated excellent performance and potential for diverse applications. In regions of research where additional data samples are limited due to the number of cities, this approach of utilizing GAN for data augmentation exhibits innovativeness and practicality. This also constitutes another focal issue to be demonstrated in this study.

This study presents a deep learning neural network classification model based on CNN. The model takes complex urban features as input data and city economic competitiveness level as the target label. The central objective of this research is to investigate the feasibility of accurately classifying the levels of economic competitiveness based on intricate urban characteristics. Simultaneously, the generative capability of GAN is employed for data augmentation to investigate whether increasing the training sample size and balancing the sample distribution can further improve the classification accuracy of CNN. In terms of experimental data, feature engineering constructed an urban feature system containing 1008 independent variables, and the experimental data used were collected from 283 prefecture-level cities in China for the period 2012 to 2019 (eight years), which resulted in a combined sample size of 2264.

### Research contributions and innovations

The primary contributions of this study are summarized as follows:

1. In this study, we constructed a complex urban feature system comprising 1008 city indicators and applied deep learning neural networks to explore the intrinsic relationships within these features. This approach expands the investigation of urban economic development disparities beyond limited influencing factors and emphasizes the comprehensive utilization of extensive urban statistical data.
2. In terms of the data structure design and model architecture selection, indicator data representing similar urban features were arranged into rows, where each row corresponded to a distinct city feature. This organization led to the construction of a well-structured two-dimensional matrix of urban feature data. A CNN architecture matching the sample dimensions was used, thus enabling hierarchical feature extraction and relationship mining through local feature learning and high-dimensional mapping, which improved the interpretability and performance of the model. Experimental results revealed that the CNN achieved classification accuracies of 93.78% and 88.44% for the 5-class and 10-class datasets, respectively.
3. To address the constraints of limited sample sizes and uneven distributions in regional disparity research, this study employed DCGAN for data augmentation and further enhanced the stability of augmentation through generated sample filtering and ensemble generation. Through these augmentation techniques, the study achieved improvements of 0.44% and 2.00% in the accuracies of the two datasets during the experiments, thereby resulting in accuracies of 94.22% and 90.44%, respectively. This indicates a wider applicability of the proposed methods to regional difference studies.

## Materials and methods

### Construction of complex urban feature system

The competitiveness of the urban economy is a multidimensional comprehensive index, and research on the factors influencing economic development is enormous; therefore, the selection of the influencing factors should start from understanding what competitiveness is. Begg [17] argues that "competitiveness" is a vague concept with two dimensions. On the one hand, it is equivalent to the "performance" of a city, which intuitively reflects the level of its economic development, and on the other hand, competitiveness is related to competition. A more competitive city is one that offers better products and services than its competitors. Based on this concept, the feature system of urban economic competitiveness should contain both direct and indirect indicators. Direct indicators are indicators of urban economic performance, which directly measure the level and scale of the urban economy. Indirect indicators are measures of the resources and services that a city can provide, which are necessary for the sustainable and high-quality development of the urban economy.

Similar to these direct and indirect indicators, another explanatory approach to understanding the concept of urban economic competitiveness distinguishes between "input" and "output" indicators [4]. The economic performance of a city, which includes factors such as residents’ income, economic structure, and local output value, is categorized as "output indicators," representing the economic development outcomes exhibited by the city. In contrast, factors such as infrastructure, city size, and education that have significant impacts on economic performance are considered "input indicators." This explanatory approach elucidates the inherent logical relationship of competitiveness, which is a complex concept, and provides feasibility for its quantification. However, excessive focus on economy may lead to a surge in environmental issues. Balancing resource conflicts between economic growth and environmental protection in urban development is the core of achieving sustainable economic development; it is also an important dimension for understanding the concept of economic competitiveness [18].

Based on the previous discussion, the construction of a complex urban feature system in this study should commence by distinguishing between direct (outputs) and indirect indicators (inputs). Considering the resource game between environmental protection and economic development, and recognizing that environmental indicators have a distinct impact mechanism compared to other indirect indicators on the economy, it is essential to separate environmental indicators as a distinct dimension. A city feature system comprising three basic dimensions: economic (direct indicators), social (indirect indicators), and environmental (indirect indicators) was formed. Within the economic dimension, macroeconomic performance, economic structure, financial performance, market size, and fiscal performance are widely utilized as foundational metrics [19, 20]. Based on this foundation, the real estate industry, which is a primary source of fiscal revenue for most local Chinese governments and plays a significant role in monetary and financial policies, was included as an indicator of local economic performance [21]. Moreover, while assessing economic competitiveness from the perspectives of social equity and sustainable development, the ultimate goal of economic development was to enhance the living standards of residents [22]. Thus, indicators related to resident income levels and employment structures also warrant attention.

In the social dimension, the supply of infrastructure, including transportation, water, gas, electricity, and the Internet, directly affects a city’s attractiveness and competitiveness [23]. Moreover, urban size determines a city’s market potential and resource scale, thereby representing a crucial factor influencing economic development [24]. In addition to hardware conditions, the high-quality development of a city’s soft power, which is represented by education and healthcare, can directly improve human capital, innovation capacity, and quality of life, which has received increasing attention [25].

In the environmental dimension, the existing research exhibits varying focal points while assessing urban environments and sustainability. These points can be categorized into three main dimensions: environmental factor provision, environmental governance capacity, and environmental quality outcomes [26, 27]. Based on these three dimensions, indicators for environmental protection facilities, pollutant emissions and disposal, and environmental quality monitoring were selected as the corresponding quantifiable indicators.

In the previous sections, the two initial levels of indicators for the urban economic competitiveness characteristic framework were determined through a literature review. Based on this, the last two levels of indicators in the feature system were identified to form a four-level indicator system. In the feature engineering of deep-learning models, providing more feature indicators can enhance a model’s generalization and representation capabilities. However, caution must be exercised when adding features—considering their representativeness and data quality as redundant or irrelevant indicators can yield adverse results [28]. Therefore, two primary factors were considered in the selection of specific indicators for this study. First, the chosen indicators must be representative and sufficiently explanatory within the scope of higher-level indicators. Second, the data quality of the indicators is of paramount importance. To increase the sample size, samples were drawn from multiple years and encompassed most of the prefecture-level cities in China. Consequently, there are stringent requirements for consistency and scope in the statistical standards of indicators. Based on the above conditions, this study aims to construct an urban feature system with more indicators to provide more effective features for model training to explore the complex inherent relationships of urban economies. Official and authoritative statistical data were prioritized during the selection process. In total, 598 indicators were selected to ensure adequacy and data quality. Among them, 410 indicators were subjected to per-capita normalization, thereby resulting in a cumulative total of 1008 specific indicators. The entire urban feature system contained 3 primary, 14 secondary, 42 tertiary, and 1008 specific (quaternary) indicators. The indicators and quantities included in the urban feature system are referenced in Table 1. Detailed indicators and their respective data sources are listed in S1 Appendix.

**Table 1:** Urban feature system.

Primary indicators	Secondary indicators	Tertiary indicators	Number of specific indicators
Economic Indicators	Basic Economic Performance	Gross regional product (GRP)	6
		Industrial Enterprise Performance	24
		Consumer Goods Market Size	12
	Economic Structure	Technology Innovation	10
		GRP Structure	6
		Sources of Fixed Asset Investment Funds	26
		Fixed Asset Investment Amount	30
	Financial Performance	Deposit and Loan Amount	12
		Digital Financial	10
	Marketization and Openness	Number of Enterprises	13
		Enterprise Output Value	22
		Foreign Trade Amount	2
	Land and Real Estate Market	Real Estate Market Investment Amount	12
		Land Market	107
		Average House Price	12
	Government Finance Structure and Operations	Financial Revenue and Expenditure Amount	18
		Number of Public Utility Employees	8
	Resident Income and Employment Distribution	Personnel Employment	24
		Employment Structure	84
		Resident Income and Consumption	8
Social Indicators	City Size	Population Size	9
		Urban Built-up Area Size	11
		Nighttime Lights Data	12
		Public Cultural Facilities	24
	Scale of Urban Construction	Telecommunications Services	16
		Water Supply Capacity	97
		Gas Supply Capacity	51
		Electricity Supply Capacity	6
	Transport Infrastructure	Passenger Traffic Volume	8
		Freight Traffic Volume	12
		Road Traffic Construction Scale	28
		Rail Transit Facilities Scale	80
	Education and Medical Service	Educational Facilities	23
		Number of Teachers	12
		Number of Students	16
		Medical Service Providers	14
Environmental Indicators	Pollutant Emission Treatment	Pollutant Emissions	10
		Pollutant Treatment Volume	60
	Environmental Facilities	Environmental Protection Facilities	32
		Urban Greening	26
	Environmental Monitoring	Air Quality Monitoring	15

### Data collection

The data for the independent variables included urban statistics for 283 prefecture-level cities in China over an 8-year period from 2012 to 2019, for a total of 2264 city samples. Some cities such as Hong Kong, Macau, and Taiwan were not included in the study. Combining the data available at the prefecture level, each city sample contained 598 original feature variables, 410 of which were selected for per capita calculation, with a total of 1008 features for each city. Most of the data were collected from the national statistical yearbooks and information publicity of national ministries and commissions.

The dependent variable, the urban comprehensive competitiveness index, was adopted from the *Annual Report on China’s Urban Competitiveness* [29] published by the National Academy of Economic Strategy (NAES), Chinese Academy of Social Sciences (CASS), and China Social Sciences Press. This report presents the economic competitiveness index of 294 cities in China using a system of indicators that includes regional output, business, finance, education, population, environment, and infrastructure. Data from 2012 to 2019 (11th–18th) reports were selected for this study.

### Theoretical foundations of deep learning neural networks

A neural network is a mathematical model that enables hierarchical processing of complex input data and is commonly used for accurate classification and prediction. The basic building units of a neural network are neurons, and multiple interconnected neurons constitute the entire network. A neuron comprises inputs, outputs, weight parameters, bias terms, and an activation function. During computation, a neuron receives input signals, multiplies them by the corresponding weight parameters, and then adds a bias term to obtain a weighted sum. This weighted sum serves as the input to the activation function, which calculates the output of the neuron. The activation function is nonlinear, thus allowing the neural network to learn complex nonlinear relationships between input data by mapping the inputs to a high-dimensional nonlinear space. This effectively enhances the expression and generalization ability of the model, resulting in a significantly improved performance in classification and prediction tasks compared to linear models. Learning capability is the core feature that distinguishes neural networks from other computational models [30]. Learning refers to the process of iteratively adjusting network parameters during training to minimize the discrepancy between the output values and the ground truth. In this process, the neural network automatically learns and extracts features from the data, ultimately minimizing the loss function and maximizing prediction and classification accuracy. This automatic solution of the optimal model parameters allows the trained neural network to be better applied to unknown datasets and environments.

### Model selection

The CNN was chosen as the analytical model for studying urban economic competitiveness in this study for the following reasons: First, studying the competitiveness of cities as complex systems, involves multiple urban indicators and their intricate interrelationships. These interactions are present not only among specific indicators, but also extend to high-dimensional indicators comprising multiple specific indicators. The efficient extraction of features from such high-dimensional feature data, considering the hierarchical nature of features, is a core influencing factor in model selection. CNNs can efficiently extract local features and spatial correlation information from the matrix data through their designs of local connections and shared weights. This spatial correlation embodied in the urban indicator matrices can be understood as the interaction between higher-dimensional indicators, which are commonly influenced by several specific indicators. With the stacking of multiple convolutional layers, the CNN progressively extracts higher-level abstract features, thereby achieving hierarchical feature extraction of the interactions among indicators from different dimensions within the urban feature system. This approach of local feature extraction and multi-level modeling aligns with the structure of the complex urban feature indicator system, thereby providing stronger interpretability of results for the research and reducing the understanding confusion and result mistrust caused by the "black box" mode.

Second, in comparison with the current common sequence neural networks, such as the recurrent neural network (RNN) and transformer neural network, it is technically feasible if the city indicators are directly used as training samples in the form of one-dimensional sequence data. However, sequential neural networks primarily capture the positional relationships of various indicators in a sequence and the dependencies between adjacent indicators, which means that the order of the arrangement of indicators in the sequence needs to be carefully considered. Theoretically proving the specific order of each indicator for urban features is challenging or even meaningless. Therefore, the interpretability of results is limited while applying such abstract data without clear ordering significance to sequence neural networks. In summary, considering the capabilities of the CNN in spatial correlation, multi-level modeling, and feature extraction, which are more aligned with the architecture of the urban feature system, it is a reasonable choice in terms of model performance and interpretability.

### CNN theory

CNNs are deep feed-forward neural networks with local connections and weight sharing. Compared with traditional neural networks, neurons in the hidden layer do not require to be connected to all the neurons in the previous layer because of the application of convolutional kernels. In addition, the application of shared weight parameters for the same set of neuron connections effectively reduces the weight parameters and accelerates model convergence. The core structure of a CNN is the convolutional layer. The convolutional kernel extracts features of the images and forms a feature map by sliding and progressively performing convolutional operations. Through multiple convolutions, the feature map size is gradually reduced, and the network depth is increased to extract more complex and abstract features from the images. The formula for the convolution calculation is

$$h_{i,j}^{\left(l\right)}=\sum_{k=1}^{m^{\left(l-1\right)}}\sum_{u=1}^{k_{w}}\sum_{v=1}^{k_{h}}w_{k,u,v}^{\left(l\right)}a_{i+u-1,j+v-1,k}^{\left(l-1\right)}+b_{i}^{\left(l\right)},$$ (1)

$$a_{i,j}^{\left(l\right)}=f(h_{i,j}^{\left(l\right)}),$$ (2)

where $h_{i,j}^{\left(l\right)}$ is the value of the feature map in row *i* and column *j* in the *l*th layer of the convolutional layer, *m*^(*l*−1)^ is the number of feature maps in the (*l*−1)th layer, *k*_*w*_ and *k*_*h*_ are the width and height of the convolution kernel, respectively, and $w_{k,u,v}^{\left(l\right)}$ is the weight value of the *k*th convolution kernel in row *u* and column *v* in the *l*th layer. $a_{i+u-1,j+v-1,k}^{\left(l-1\right)}$ is the value of the *k*th feature map at row (*i*+*u*−1) and column (*j*+*v*−1) in the (*l*−1) layer. $b_{i}^{\left(l\right)}$ is the bias term of the *i*th feature map of the *l*th layer, and *f* is a nonlinear activation function. A typical CNN generally contains a pooling layer, which includes average and maximum poolings, to reduce the size of the feature map and thus increase the computational efficiency. A fully connected layer is commonly used as the final output layer, which maps the feature information from the previous layer to the next layer and converts the feature map into a one-dimensional vector for final classification and recognition.

### Data augmentation

CNNs have powerful capabilities for data feature extraction and classification; however, these capabilities are based on large volumes of training samples [31]. Insufficient training samples would result in decreased accuracy or overfitting, which is often characterized by an increase in the loss value and a slight increase in accuracy or a constant. This indicates that the model overlearns meaningless features, resulting in a lower generalization ability. In this case, the model has higher accuracy on the training set and lower accuracy on the test set. Typical approaches to improve the generalization ability include dropout [32], migration learning [33], and pre-training [34]. Although these approaches have been widely applied, they mainly target the data structure of image samples and have certain requirements for the sample size, which are less compatible with this research in terms of the size and distribution of samples.

Many studies have demonstrated that increasing the training sample size can significantly improve model performance [35]. Common data enhancement methods are cropping, stitching, and flipping of original data. Sharpening, blurring, and color adjustment can also be applied when processing image data. These treatments directly change the data values of the samples; however, for image samples, they do not modify the objects on the screen; instead, they highlight the features more clearly. By contrast, abstract data for economics do not depict concrete objects; therefore, the application of these approaches is limited.

The employment of GAN in data augmentation tasks is a more focused approach. Generators constantly learn and integrate features in competition with discriminators and create new features in the process, which can be considered as a process of unlocking additional information in the training data [36]. Yi et al. [37] found that applying a GAN for data enhancement in medical image detection could provide rich and convincing features, further improving the model accuracy. Lim et al. [38] addressed the data imbalance problem using data generated by a GAN, which effectively improved the recognition rate of a detection model. In this research, the number of city samples was fixed; however, the economic development level of Chinese cities has apparent unevenness, that is, a few municipalities and provincial capitals are economically developed, whereas most cities have a low level of economic development. Limited sample sizes and uneven distributions often occur in social science research. Therefore, using a GAN for training sample expansion provides richer features compared to the original samples and improves the generalization ability of the models. In addition, sample expansion balances the data distribution and prevents the CNN from favoring the data-rich category in the classification, thereby improving the model classification accuracy.

### GAN theory

A GAN is a network framework consisting of two deep neural networks: a generator that learns the distribution of real data and generates similar data and a discriminator that distinguishes whether the input data are real or generated. The training of generators and discriminators is a zero-sum game in which the maximization of one party’s gain must be achieved by minimizing the other party’s gain. The two models compete against each other in alternate training, aiming to generate samples that are similar but difficult to distinguish from the real samples. Considering that GAN provides a model framework, multiple variants of GAN have been implemented by replacing the discriminator and generator models and adding other processors. Radford et al. [39] constructed the DCGAN utilizing CNNs as generators and discriminators, which demonstrated an impressive performance in data classification and high-quality image generation. Mirza and Osindero [40] proposed a conditional generative adversarial network (CGAN) that imposes constraints on the generated data by adding labeling variables, such that the GAN is controlled to generate samples with specific requirements. Odena et al. [41] proposed an auxiliary classifier GAN (ACGAN), which is a multicategory classification network added to the original structure of CGAN. In ACGANs, the generated data are simultaneously used in the training of the discriminator and classification network, preventing the training of the classification model from crashing caused by unbalanced sample distribution, thereby expanding the applications of GANs. The value function formula of a GAN is

$$\underset{G}{\min}\;\underset{D}{\max}V(D,G)=E_{x∼P_{data}(x)}[\log\;D(x)]+E_{z∼P_{z}(z)}[log\;(1-D(G(z)))]$$ (3)

where *E* is the expectation, *D* is the discriminator, and the output of *D* ranges from 0 to 1, which represents the probability of generating real input samples. *data* represents the real data used for training, *G* is the generator, and *z* is a noise variable input to *G* for data generation. $\underset{G}{min}$ represents the objective of *D* is to maximize the value function *V*, which is equated to maximize *D(x)* and *1-D(G(z))*. Therefore, the training objective of *D* is to discriminate the real samples as real (Output 1) and the generated samples as false (Output 0). Similarly, the objective of *G* is to minimize the value function, which is equivalent to minimizing *1-D(G(z))*, such that the training objective of *G* is to deceive *D*, prompting *D* to discriminate the generated samples as real (Output 1).

### Overall design of the experiment

**Fig 1:**
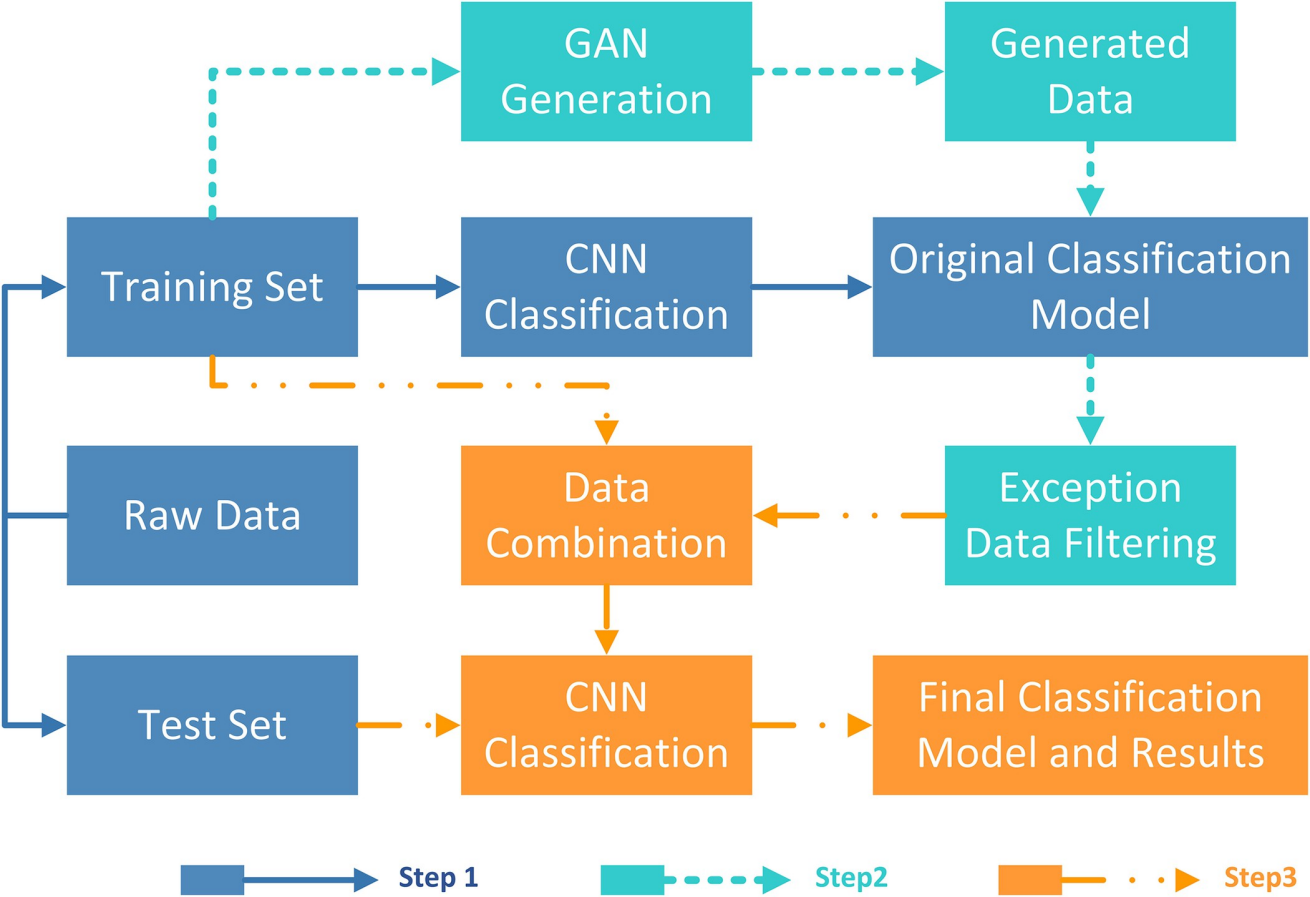
Experimental procedure.

Referring to the flowchart in Fig 1, the entire experimental process can be divided into three parts:

Step 1: The training and test sets were used for the CNN training, and an original classification model was obtained.

Step 2: The training set was applied to the GAN training for categorical data generation, and the generated data were filtered using the original classification model to eliminate data whose classifications differed from the generated categories.

Step 3: The filtered data were combined with the training set from the raw data to form a new training set, and the CNN was used again for training to obtain the final classification model.

### Architectural design of neural network models

#### Input data structure

A CNN captures the local features and spatial relationships of data through convolutional kernels. The data structure is a core factor affecting the performance of a CNN. One- and two-dimensional CNNs are common and widely used CNN structures. One-dimensional CNNs are often employed to process time-series data, the core of which extracts the sequential dependencies of series data, and thus not applicable to urban feature analysis. Two-dimensional CNNs focus on local features in the data and analyze and represent these data features through multiple extraction and condensation processes. Therefore, in this study, city feature data were mapped to the distribution of images to adapt to the structure of a two-dimensional CNN.

The feature data for each city contained of a one-dimensional vector with a length of 1008. To meet the input requirements of the network, the vector was expanded to a length of 1024 by zero padding. Subsequently, the vector was transformed into a 32×32 two-dimensional matrix. This data structure enables the network to effectively extract the spatial relationships between local and global features, maximizing the performance of the model. Therefore, by applying zero-padding and dimension transformation operations, the original one-dimensional feature vector is transformed into a two-dimensional matrix structure suitable for two-dimensional CNNs. It is better to understand this data structure as a "feature image", as an image is composed of pixels, while a feature image is composed of urban features.

Regarding the application of abstract data to CNN, the arrangement of indicators in the input data is an important aspect of feature engineering. The data structure formed by different arrangements significantly affects the capture of interrelationships between indicators and feature extraction, which is ultimately reflected in the model’s accuracy and generalization capability [42]. In a CNN, the convolutional layers extract local spatial features by sliding convolution kernels. If indicators of the same type are grouped in the same region to represent a specific feature, the convolutional kernels can capture the local features of this region more easily. This organization is also conducive to generating feature mappings that are purer and focused on specific features. In contrast, if indicators are not categorically arranged but are randomly distributed, the feature extraction process of convolution kernels becomes difficult, and chaotic feature maps would lead to poor model performance [43].

**Fig 2:**
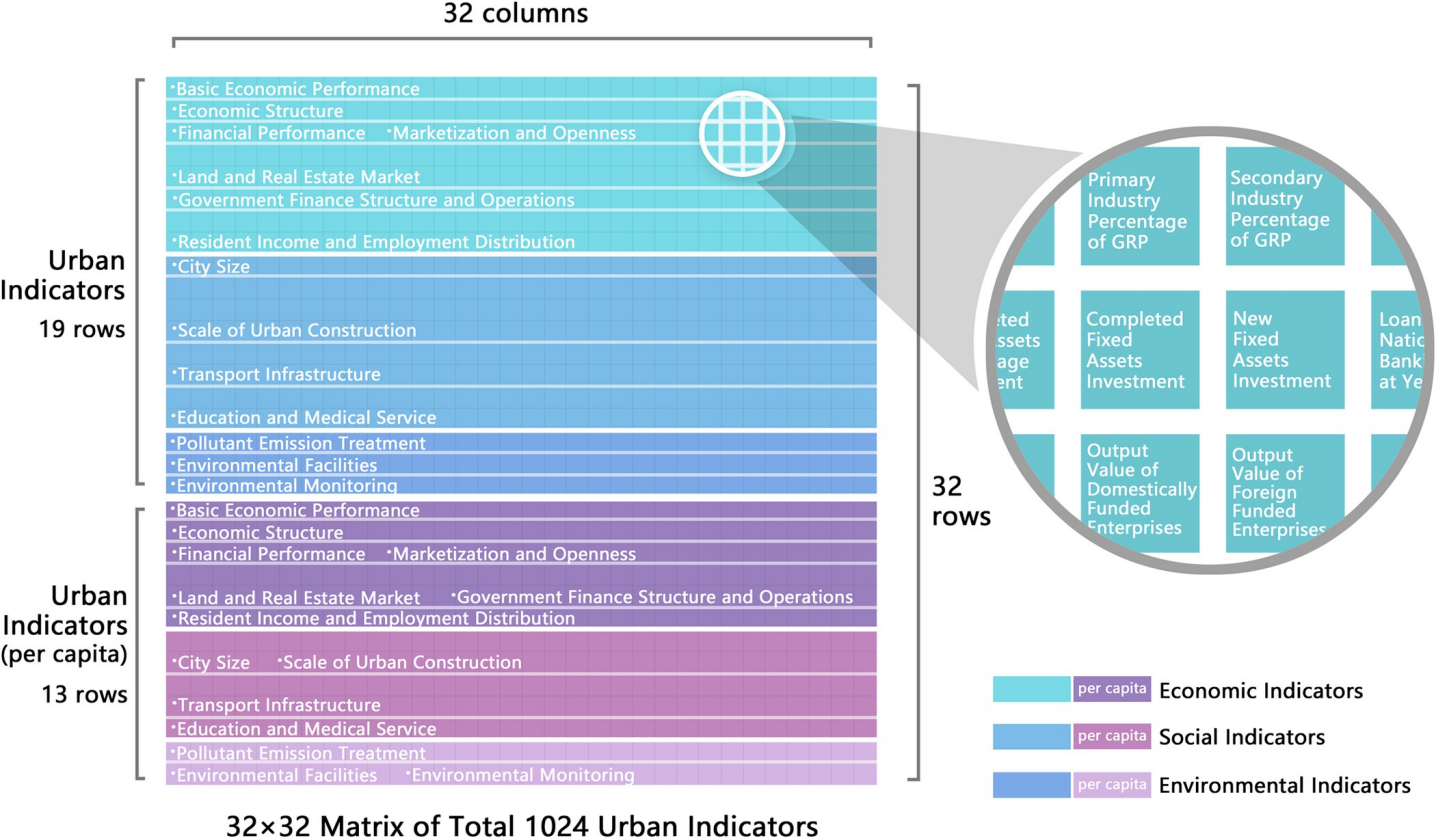
Input data structure of CNN.

In this study, a 32×32 two-dimensional matrix of the input data was divided based on row units, where indicators representing similar types of urban features were arranged in the same row. As shown in Fig 2, the data structure was organized such that the original indicators and indicators computed on a per-capita basis were initially divided and arranged in the first 19 and subsequent 13 rows, respectively. Subsequently, based on the hierarchical structure of the urban feature system, the indicators were further categorized into economic, social, and environmental indicators from top to bottom. For example, economic indicators occupied the first eight rows, with basic economic indicators in the first row, followed by a similar arrangement. Through this arrangement, the indicators representing the same high-dimensional features were grouped into the same region.

In the low-level convolutional layers, convolutional kernels can further emphasize the local features corresponding to identical region indicators and features of the same type by capturing intrinsic relationships among features of the same type. In the high-level convolutional layers, with an increase in the number of channels and a reduction in the size of feature maps, convolutional kernels can capture the intrinsic relationships among higher-dimensional features. This indicator arrangement allowed for a gradual transition from specific to high-dimensional indicators in capturing intrinsic relationships, thus providing stronger model interpretability and performance improvement compared with random indicator arrangements. In this study, a CNN was employed and the data were used to test two modes of indicator arrangement: random and categorical. When only the arrangement of the sample features was altered, the categorical arrangement led to an improvement in the model accuracy of approximately 0.89% to 1.11%.

#### CNN architecture design

The architectural design of a CNN should be adjusted based on the data dimensions, sample size, and task complexity. Excessive parameterization can lead to training difficulties and overfitting, whereas inadequate parameters can result in insufficient model expression. Given the relatively small size of the urban feature matrix (32×32), overly deep convolutional layers can result in excessively abstracted features and subsequent overfitting. Therefore, a shallower architecture with four convolutional layers was selected. Considering the limited original sample size (2264), the number of channels in the feature maps was set to 512 to ensure a sufficiently rich feature representation. In the first three convolutional layers, a 4 × 4 kernel size was chosen to capture larger local features, while remaining smaller than the local feature dimensions of the economic, social, and environmental aspects, thus preventing the extraction of overly abstract and complex local features. A stride of 2 was used to downsample the feature map dimensions by half in each layer, thus allowing the hierarchical extraction of features in higher dimensions. The convolutional layer design also includes batch normalization and dropout layers. Batch normalization layers standardize the input feature maps to reduce the internal covariate shift and improve the model convergence speed; they were added to all convolutional layers except the first. The dropout layers randomly drop a certain ratio of neurons for regularization and improve the model generalization; their ratio was set to 50% in the experiments.

**Fig 3:**
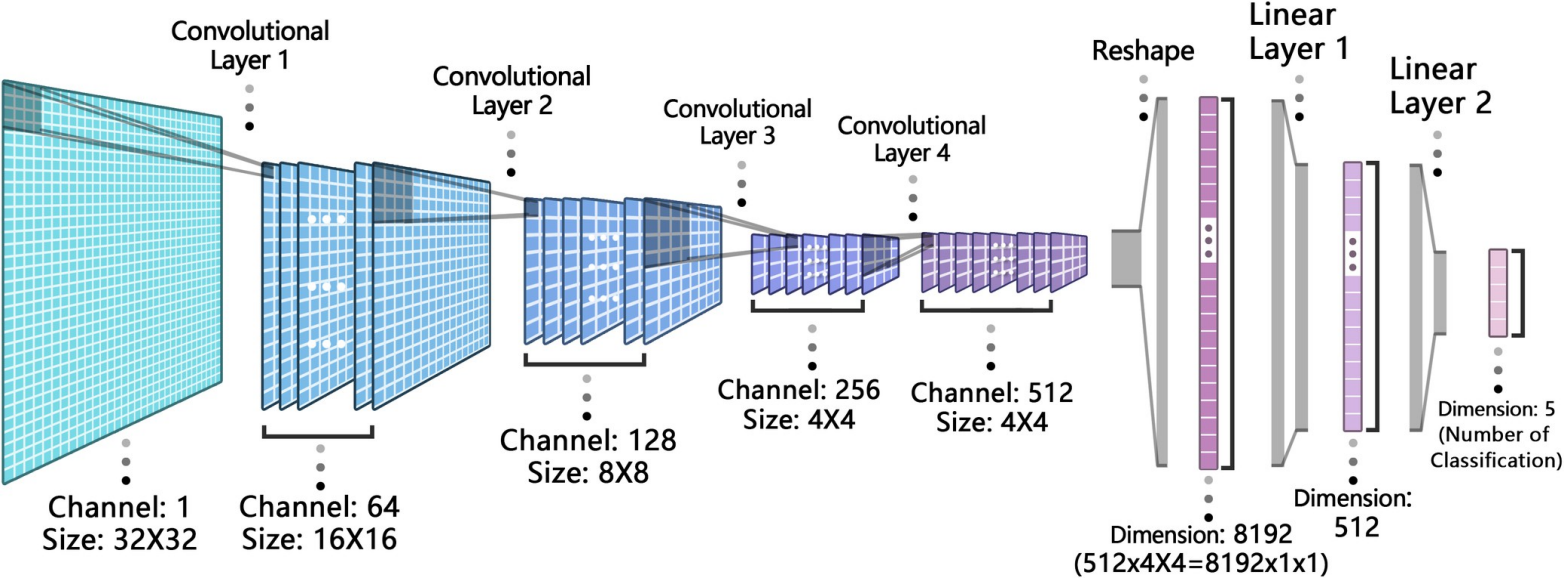
CNN model architecture.

Amongst the activation functions, we selected the leaky rectified linear unit (LeakyReLU) for all layers except the output layer. Compared to the rectified linear unit (ReLU) function, the LeakyReLU function introduces a small gradient on negative inputs, thus effectively mitigating the issue of gradient vanishing caused by neuron inactivity for negative inputs in the ReLU function and enhancing the expressiveness of the model. The output layer of the model selected the logarithm of the softmax (LogSoftmax) activation function, which is commonly used for multi-class categorization tasks. The LogSoftmax function calculation involves computing the logarithm of the output of the softmax function and converting the probability distribution into a logarithmic probability distribution. Thus, the probability calculation changes from multiplication to addition, which avoids the underflow caused by a large number of values that are significantly small and breaks the calculation accuracy limit. The negative log-likelihood loss (NLLLoss) function was chosen as the loss function. It was coupled with the LogSoftmax activation function. This combination aids in intuitive calculation of the loss for multi-class classification tasks, thereby reducing numerical stability issues and enhancing the training efficiency and convergence speed. It is important to note that the pooling layers were not incorporated into the CNN in the experiment because downsampling of the feature map was achieved by the cooperation of the convolutional kernel with the step size, and the incorporation of the batch normalization and dropout layers can prevent the occurrence of overfitting to a certain extent. Therefore, considering the limited number of urban feature metrics, the pooling layers were removed from the CNN design to prevent the loss of major features owing to the pooling layer. Fig 3 depicts the specific architecture of the CNN.

#### DCGAN architecture design

**Fig 4:**
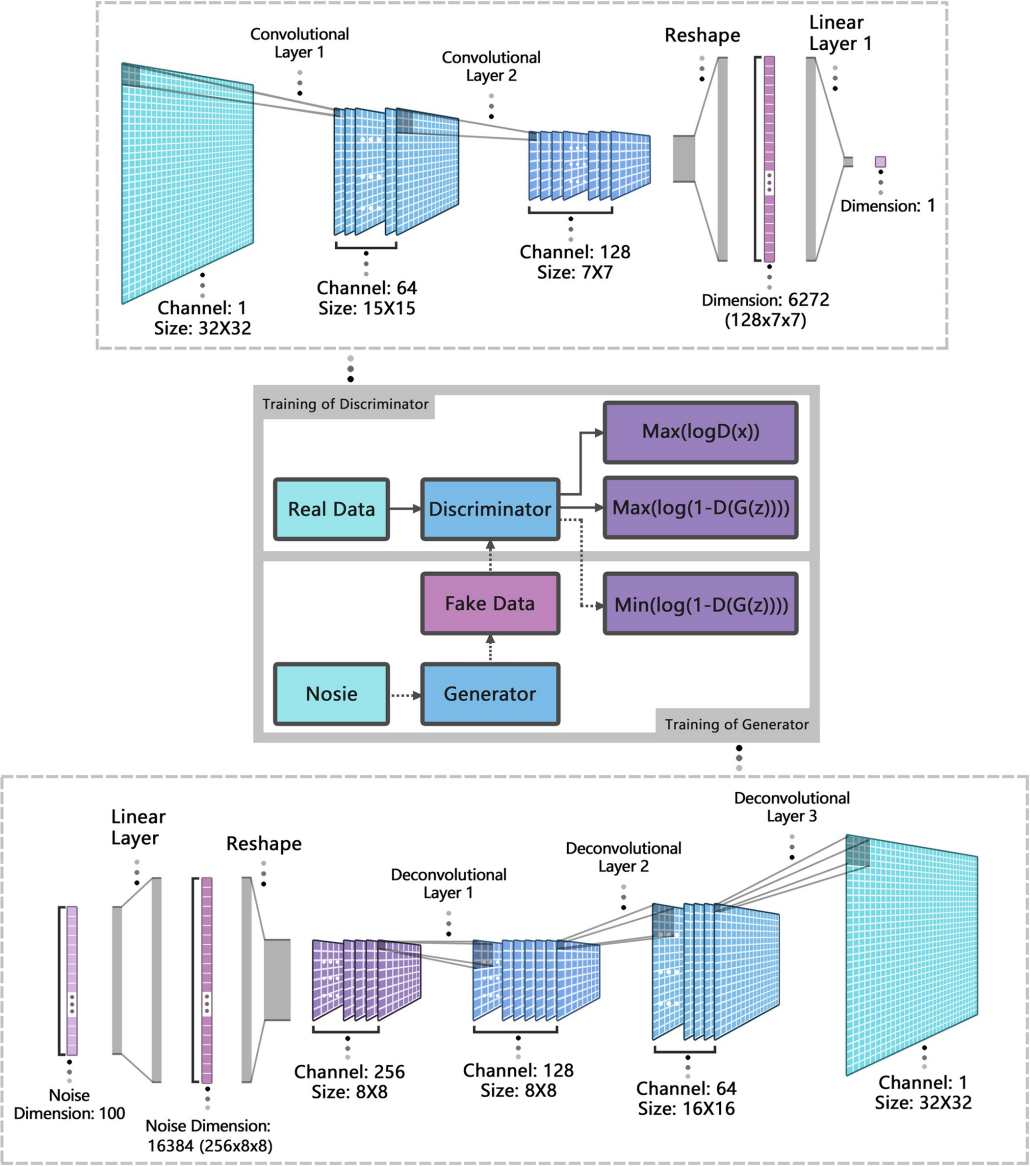
DCGAN model architecture.

A GAN is a neural-network framework in which the discriminator and generator are independent neural networks. The discriminator has a clear objective function, which distinguishes real from fake data, whereas the generator’s objective function generates samples that could be as real as possible. Thus, the generator must learn complex probability distributions, resulting in a higher training difficulty. In the model designs and hyperparameter settings of GANs, a balanced growth of the generator and discriminator capabilities is the key to successful training. Both the generator and discriminator of a DCGAN utilize a CNN. Thus, the key to the balanced growth of the two modules lies in the performance of the convolutional layers, which used a more powerful 3-layer convolutional network with a maximum of 256 feature map channels for generator. By contrast, the discriminator used a 2-layer convolutional network with 128 feature map channels. In addition, the size of the convolutional kernel of the discriminator differred from that of the generator to increase the network complexity. The specific architecture of DCGAN is referenced in Fig 4. In terms of the learning rate, the generator learning rate was set to 0.0001, which was larger than the discriminator learning rate of 0.00001, to prevent the discriminator from converging too fast and the generator training from crashing. In the convolutional layer activation function, the discriminator employed a LeakyReLU to avoid gradient vanishing, whereas in the generator, a ReLU with a stronger non-linear expressive power was used to better preserve and enhance the features of the generated data.

The generator used a hyperbolic tangent (Tanh) function as the activation function of the output layer. The output values of Tanh were within the range [–1,1] and the mean value was approximately zero, which could effectively control the range of the generator output values. In addition, benefiting from the large gradient of the output value, Tanh could simulate the data characteristics of the original sample as much as possible. The discriminator in a DCGAN is a binary classification CNN, which often uses a sigmoid activation function for its output layer. This is because the sigmoid function has a larger gradient when its input is close to zero, which makes it more sensitive in discriminating the generated data. Furthermore, the binary cross-entropy loss (BCEloss) function was adopted as the loss function of the DCGAN, which was specifically designed for binary classification tasks. It can effectively measure the label difference between the generated and real samples.

### Data preprocessing and analysis

The indicator system involved multiple units of measurement and orders of magnitude, and normalization was used to standardize the data to eliminate the influence of different dimensions on the analysis. The formula is

$$x_{norm}=\frac{x-x_{min}}{x_{max}-x_{min}}$$ (4)

where *x* represents the raw data, *x*_*norm*_ represents the data after normalization, and *x*_*max*_ and *x*_*min*_ are the maximum and minimum values, respectively, in the same dimension as the data. Initially, the normalization process was performed successively in the dimensions of the features and samples, and the data were compressed to an interval of [0,1 in two dimensions. Subsequently, a typical standardization process for CNN was applied in the feature dimension.

$$x_{std}=\frac{x_{norm}-0.5}{0.5}$$ (5)

where *x*_*std*_ is the data after standardization, which was distributed over a range of [–1,1].

Sample labels were processed through equal-width binning to divide the continuous variables of the original data into discrete variables so that an increase in the sample size for each category could effectively improve the model accuracy and efficiency. This feature transformation technique is widely used in machine learning [44]. First, the raw urban comprehensive economic competitiveness index data were standardized to an range of [0,1], after which the data were divided into five and 10 classes. The division of five classes used a width of 0.2: Label 1 represents lowest competitiveness and Label 5 represents highest competitiveness. The division of 10 classes used a width of 0.1.

The sample size of each class listed in Table 2 shows that the distribution of the sample was highly unbalanced, and a large proportion of cities had a lower level of economic competitiveness, which is the same as the current development status of Chinese cities. China’s southeastern coastal region has obtained more development opportunities by benefiting from open policies, natural environment, and large ports. In addition, to prevent resource outflow, provinces generally focus on developing provincial capitals; however, excessively strong provincial capitals tend to siphon resources from neighboring cities, resulting in a greater urban development gap.

**Table 2:** Sample size of each classification.

Five-class	Competitiveness class	1 (Lowest)		2		3		4		5 (Highest)	
	Sample size	1751		405		81		15		12	
**Ten-class**	**Competitiveness class**	1	2	3	4	5	6	7	8	9	10
	**Sample size**	1209	654	199	97	54	21	12	6	2	10

In this type of data distribution, basic data enhancement of the raw samples was achieved through reverse sorting of the feature data and doubling the original samples to a total sample size of 4528. The samples were randomly divided into training, validation, and test sets at a ratio of 8:1:1, with sample sizes of 3628, 450, and 450, respectively.

### Evaluation metrics

The classification accuracy of the validation and test sets was selected as the main evaluation index of the CNN performance. Accuracy is the ratio of the samples with the same predicted labels to the original labels in the total samples; the higher the accuracy, the better the model performance. Additionally, the output value of the cross-entropy loss function and speed of convergence assisted in the evaluation. An early stopping strategy was adopted in the training, which indicates that the training would be terminated when the loss value and the accuracy of the validation set are leveled off. To precisely compare the model performance before and after data augmentation by the DCGAN, all the CNN trainings in the experiment had a learning rate of 0.0001, a training set batch size of 1024, and a validation set batch size of 450.

The experimental equipment was equipped with a Windows 11 operating system and an Nvidia GeForce RTX 3070 graphics card. The neural network model was built using Python 3.9.12 and PyTorch 1.12.0.

## Results

### Classification of original dataset using CNN

**Fig 5:**
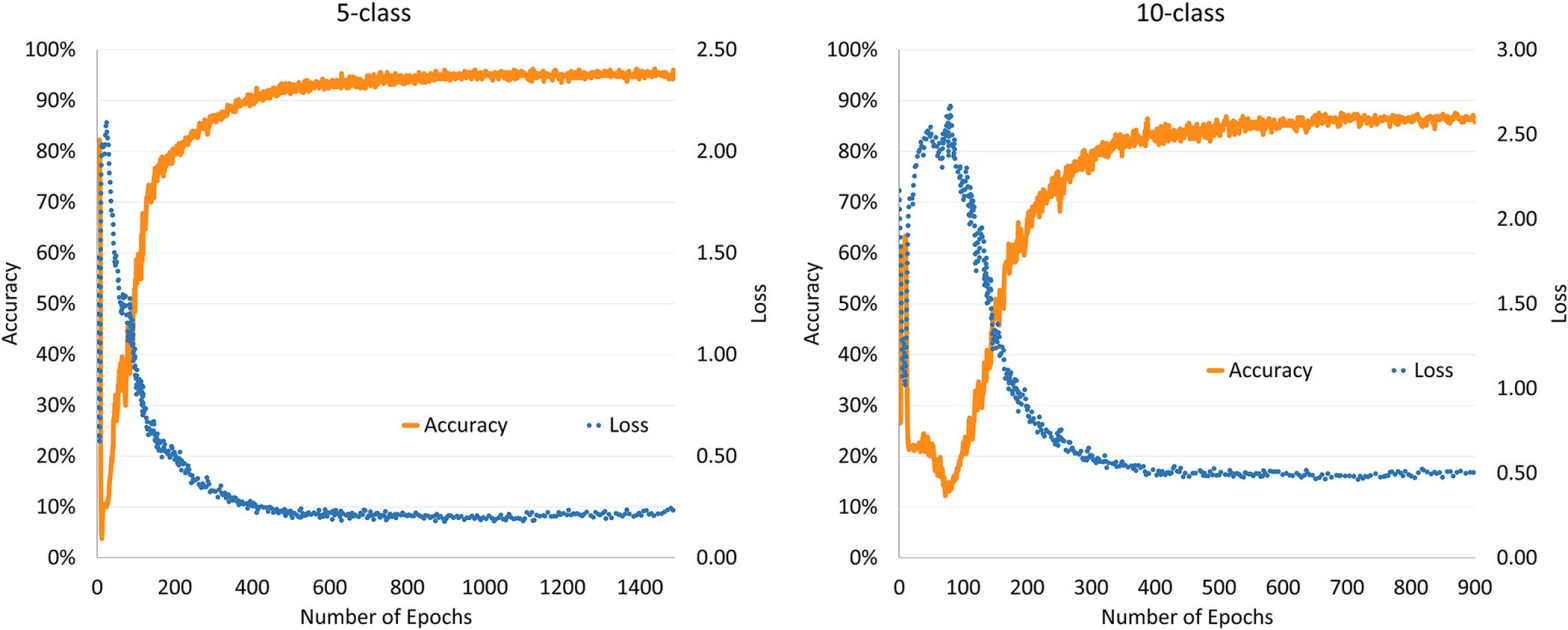
Classification accuracy and loss values of the original dataset.

This experiment was the first to evaluate the performance of a CNN in accurately classifying urban economic competitiveness. As shown in Fig 5, in the training using the 5-class original dataset, the validation accuracy and loss values stopped improving at approximately 1400 epochs, indicating that the model converged. The model achieved a maximum accuracy of 96.44% in the validation test. In addition, the trained model classified the test set with an accuracy of 93.78%. The model achieved faster convergence rate using 10-class samples, and the variation in the validation performance metrics leveled off at approximately 900 epochs, at which time the accuracies of the model using the validation and test sets were 89.11% and 88.44%, respectively. The results showed that the model exhibited high accuracy rate in the 5-class test and less accurate in the 10-class test because the difficulty in classifying increased as the number of classes increased. The model performs well in terms of accuracy, which proved the effective utilization of the CNN and urban economic characteristic system data. In addition, the model demonstrated similar classification accuracy for both the validation and test sets, indicating that the model can adapt to data changes and has good generalization ability.

### Data augmentation using DCGAN

#### Data generation

The experiment also evaluated whether the training sample expansion achieved by the DCGAN could improve the performance of the CNN. The original samples of each class were placed separately in the DCGAN for generative training. To maintain balanced growth of the discriminator and generator performances, the learning rates were set to 0.00001 and 0.0001. A lower learning rate set for the discriminator prevents training crashes caused by rapid growth in its performance. The training set batch size was 1024, and the number of training epochs ranged from 10,000 to 40,000 because of the different training difficulties caused by the different class sample sizes.

**Fig 6:**
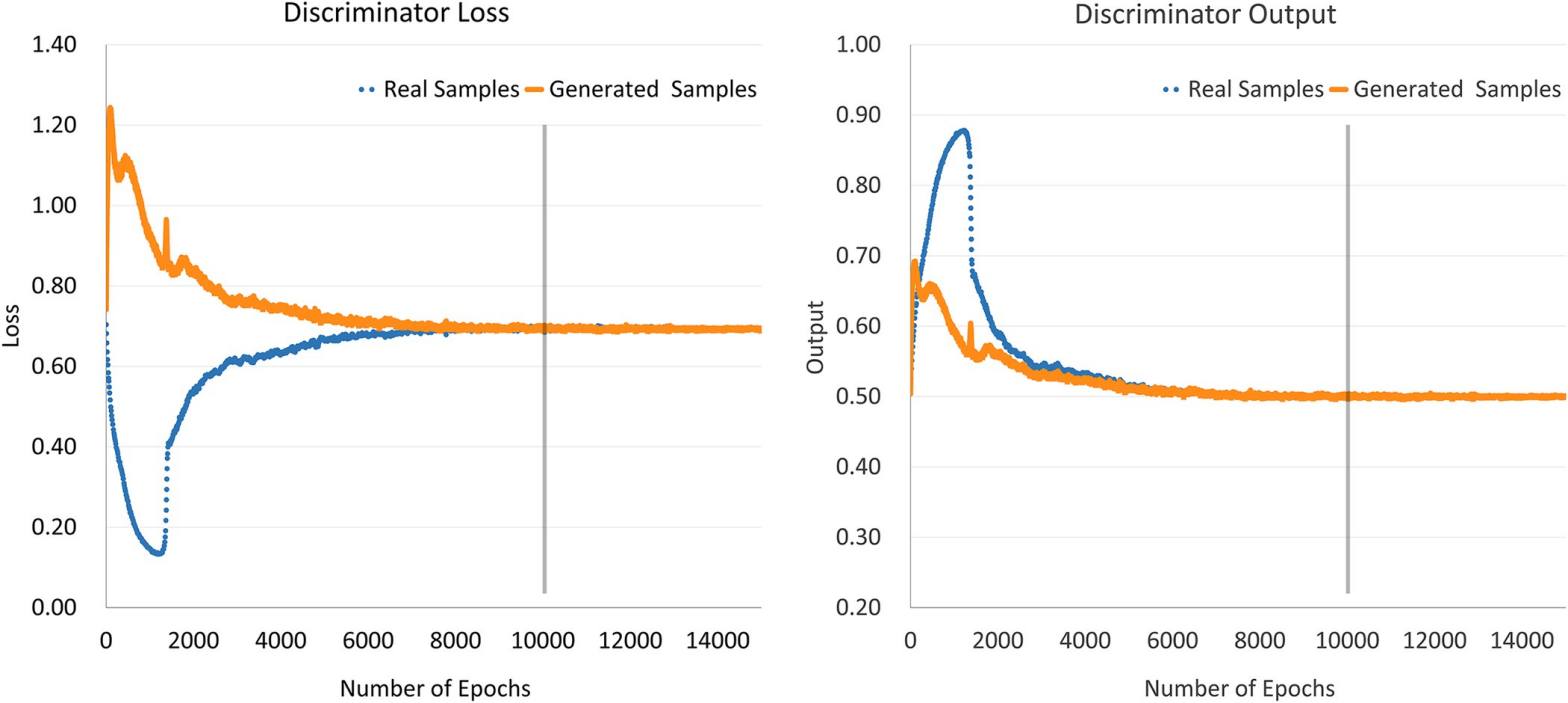
DCGAN discriminator loss and output values.

In contrast to processing intuitive data, such as images, where DGAN training can be determined by directly observing the quality of the generated images, in the case where the samples are abstract data, the progress of the model training can be determined through discriminator loss and output values. In the case where a discriminator and generator play a binary zero-sum game, the global optimal solution is that the discriminator cannot distinguish between the generated and real samples, which indicates that the output values of the discriminator for both types of samples are approximately 0.5, whereas the discriminator loss values for both types of samples converge and remain smooth. As demonstrated in Fig 6, for the training process of samples with label value 1 in the 10-class dataset, the model converges around 10,000 epochs, at which point the generated data reaches high quality.

#### Data filtering

In data augmentation tasks, the trained GAN can adopt the ensemble generation mode for data generation, which indicates that the data are generated from multiple trained models instead of employing only the best model. In the case of a large amount of data generated by a single model limited by the singularity of the model, the high similarity of the generated samples leads to a reduction in effective features. In contrast, ensemble generation is more beneficial for maintaining sample diversity [45].

The ensemble generation contained multiple models, and to prevent the defective model from generating anomalous samples, the CNN model that was trained in previous experiments filtered the generated samples, that is, it eliminated the generated samples whose expected labels were different from the CNN-predicted labels. The abnormal sample proportions of the different classes ranged from 1% to 40%.

#### Sample combination

Another factor for data enhancement of DCGAN is the number of samples that should be generated. This study tested three ratios between the original and generated samples in the enhanced dataset:

1. Large sample size (generated sample size exceeded 50,000; total sample size exceeded 60,000)
2. The total sample size was balanced for each class (each class in the augmented dataset had the same sample size, with a total of approximately 20,000).
3. The generated sample size was balanced for each class (with approximately similar generated sample size for each class)

The results of the classification accuracy test show that the model exhibited better performance only on the last combination of the augmented dataset in comparison to its performance using the original dataset. Based on the criterion, 13,700 samples were generated for the 5-class samples test, and 3,341 abnormal data points were excluded after CNN filtering, with the remaining10,359 generated samples for the final augmented dataset of 13,987 (combining the available generated dataset with the training set of original samples). In the 10-class samples test, the augmented dataset size was 13,590. Table 3 presents the specific sample quantities and allocation methods of the original dataset and augmented dataset in the experiment.

**Table 3:** Sample size of each data set.

	Original samples				Generated samples			
Classification	Original dataset	Training set	Validation set	Test set	All generated dataset	Anomalous samples	Available generated dataset	Augmented dataset
Five-class	4528	3628	450	450	13700	3341	10359	13987
Ten-class					14400	4438	9962	13590

#### Classification of augmented dataset

**Fig 7:**
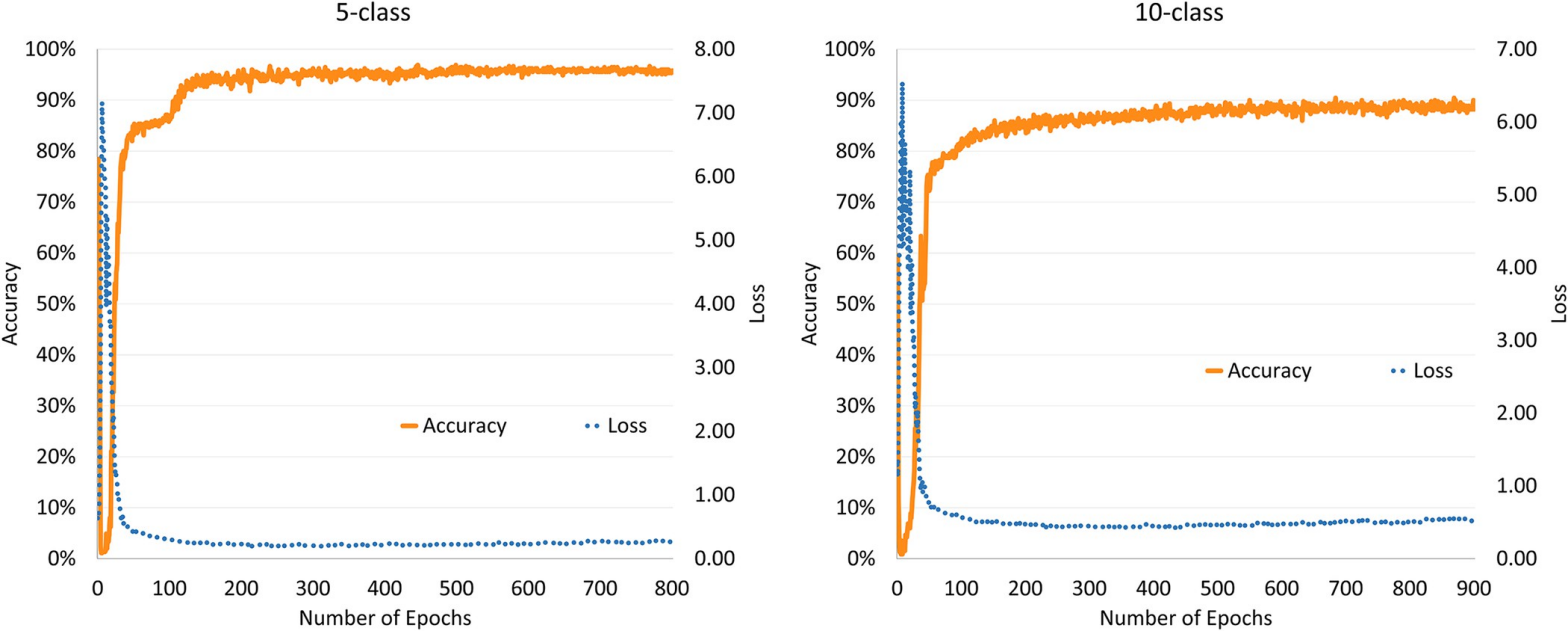
Classification accuracy and loss values of the augmented dataset.

After applying the enhanced dataset, the accuracy of the 5-class test set improved from 93.78% to 94.22%, which represents an increase of 0.44%. A more dispersed data distribution of the 10-class samples enhanced the advantages of the augmented dataset, wherein the accuracy of the test set improved from 88.44% to 90.44%, which represents an increase of 2.00%. Both samples exhibited higher accuracy rates than the original samples, thereby proving the validity of the enhanced dataset. Meanwhile, as shown in Fig 7, the convergence rate with the augmented dataset is significantly faster than that with the original dataset. The specific accuracies of the original dataset and augmented dataset are referenced in Table 4.

**Table 4:** Classification accuracy.

Dataset	Classification	Original dataset	Augmented dataset	Accuracy improvement
Validation Set	Five-class	96.44%	96.89%	0.45%
	Ten-class	89.11%	90.44%	1.33%
Test Set	Five-class	93.78%	94.22%	0.44%
	Ten-class	88.44%	90.44%	2.00%

#### Quality assessment of generated data

Verifying the quality of generated data is crucial for methods that use GANs to generate augmented data. The generated samples were employed as training data for subsequent CNN training. Low-quality samples introduce bias and reduce the model’s generalization capability and accuracy. Furthermore, insufficient diversity may lead to the failure of the data augmentation method. Owing to the lack of a standardized evaluation method and criteria, assessing the quality of data generated by GANs has consistently proved to be challenging. In the widely applied field of image generation using GANs, inception score (IS) and Frechet inception distance (FID) are representative evaluation standards. These methods utilize inception networks pre-trained on the ImageNet dataset to compute the distances between the generated and original data, based on features. Because they rely on pre-trained networks, the application of these methods requires the generated samples to be concrete images, the categories of which are included in ImageNet. These methods are not applicable to the abstract data used in this study. To address this situation, an evaluation method based on the performance of the generated data in downstream tasks was proposed [46]. This method does not calculate the feature distances between the generated and original data, but instead, applies the generated data to specific tasks and evaluates the quality and diversity of the generated data based on its actual performance in these tasks. This evaluation approach is more suitable for abstract data. Specifically, the evaluation of data quality included two tests: GAN-train and GAN-test. The GAN-train utilizes the generated data to train a CNN, and then evaluates the model performance using the original data as the test set. Because the CNN can only learn features from the generated data during the training process, a higher G-train classification accuracy signifies that the generated data can provide more diverse features and better data quality. In GAN-test, the CNN is trained on the original data and tested on the generated data. Higher accuracy indicates higher quality and realism of the generated data. Both GAN-train and GAN-test were tested for diversity and quality, and the performance of the generated data in these two aspects was considered to be a core factor in achieving data augmentation.

In terms of the evaluation criteria, the evaluation of the GAN-test is relatively straightforward, wherein the difference in accuracy of the original and generated data are compared, when used as the test set. In the experiments, when the generated data matched the sample quantity of the original dataset, the accuracies of the generated data in the 5-class and 10-class tests were 89.20% and 87.48%, respectively. These accuracies when compared to the original dataset accuracies of 93.78% and 88.44%, respectively, revealed a close match and indicated high data quality in the generated samples. However, assessing the GAN-train is more complex because of its use of generated data for training, thus leading to an infeasible direct comparison with the accuracy of the original dataset. Therefore, the evaluation involved a comparison with the accuracies of methods similar to those in existing studies. Referring to the original paper proposing this evaluation method, DCGAN achieved 65.0% GAN-train accuracy on the Canadian Institute for Advanced Research 10 (CIFAR-10) dataset when using the same number of generated and real samples [46]. In another study, using a similar evaluation method, DCGAN generated 1000 samples from a tomato leaf disease dataset with 240 samples. When both the generated and real samples were used jointly for GAN-train, the accuracy reached 66.00%. In addition, the authors comprehensively analyzed other evaluation methods and concluded that the quality and diversity of the generated data were as expected, when the GAN-train accuracy was 66.00% [47]. In this study, when the development set sample size matched that of the original dataset, the GAN-train accuracies for the 5-class and 10-class samples were 79.31% and 55.32%, respectively. When the development set sample size was increased to three times that of the original dataset, the accuracies changed to 78.89% and 65.06%, respectively. Notably, the 10-class samples showed increased accuracy after incorporating additional generated samples for training, whereas the accuracy of the 5-class samples remained relatively stable. A comprehensive comparison of the GAN-train accuracy in the two aforementioned studies revealed that the 5-class samples demonstrated superior data quality and diversity, whereas the 10-class samples exhibited slightly inferior performance, although it was satisfactory. Table 5 displays the different datasets used for GAN-train and GAN-test, along with their respective test accuracies.

**Table 5:** Accuracy of generated sample quality assessment.

Evaluation method	Development set data source	Test set data source	Classification	Development set sample size	Test set accuracy	Development set sample size	Test set accuracy
GAN-train	Generated samples	Original samples	Five-class	4528 (1 times the original dataset)	79.31%	13584 (3 times the original dataset)	78.89%
			Ten-class		55.32%		65.06%
GAN-test	Original samples	Generated samples	Five-class	4528	89.20%		
			Ten-class		87.48%		

### Robustness analysis

A total of four validation methods were designed to validate the combined performance of the research methods. First, to verify the performance of the custom-designed CNN (CNN-Custom), the accuracy was compared while using very deep convolutional networks with 11 layers (VGG11) and very deep convolutional networks with 16 layers (VGG16) on the same dataset. Second, to validate the influence of the intrinsic relationships between different data structures and indicators on the model, two neural networks: vision transformer (VIT) and long short-term memory (LSTM), which differ from the CNN architecture, were selected for validation. Third, by constructing regional feature data for China’s county-level administrative areas, the stability and generalizability of the CNN-Custom architecture were verified. Fourth, in terms of data augmentation, a method similar to the experimental design rationale, ACGAN, was used to validate the effectiveness of the data augmentation method.

#### Performance comparison

VGG, a classic CNN architecture, has gained strong universality and interpretability owing to its concise structural design and deep network architecture. It is frequently used as a performance benchmark in CNN research. Compared to CNN-Custom, VGG has a deeper network structure, with VGG11 and VGG16 having 8 and 13 convolutional layers, respectively. The additional convolutional layers in VGG enhance its feature extraction capabilities; however, this augmentation also increases the possibility of overfitting. Because VGG requires fixed 224×224 data dimensions, the data dimensions were expanded to the required size by zero-padding. The experimental results revealed that both VGG11 and VGG16 achieved an accuracy similar to that of CNN-Custom on the validation set; however, they provided a slightly inferior accuracy to CNN-Custom on the test set with 10-class samples. Meanwhile, VGG16 exhibited an inferior performance to VGG11 (test set: 5-class—VGG11: 94.89%, VGG16: 94.00%; 10-class—VGG11: 87.11%, VGG16: 87.11%). This phenomenon can be attributed to the excessively abstract features extracted by the deeper convolutional layers of the VGG concerning the number of features within the urban feature system and data volume. This abstraction can lead to overfitting. Furthermore, the higher model complexity of VGG16 and the lower data volume for the 10-class samples exacerbated this occurrence.

#### Data structure and feature relationships validation

Considering urban feature data as abstract information curated manually and containing various potential data structures and intrinsic relationship patterns among indicators, a ViT and a LSTM were chosen as comparative models. This selection aimed to validate the efficacy of the proposed approach for hierarchical urban feature extraction based on CNN local feature extraction and high-dimensional feature mapping. ViT is an emerging computer vision model that uses the same input data structure as a CNN. By contrast, ViT divides the data matrix into patches of equal size via convolutional operations and models the global relationships between patches through a self-attention mechanism to capture data features. In contrast to the emphasis of CNNs on local feature extraction, ViT focuses more on holistic feature representation through global feature encoding. In the experiments, ViT used a 4 × 4 patch size, an embedding dimension of 256, 12 encoder blocks, 8 attention heads per block, an MLP ratio of 2, and a representation size of 256. The experimental results revealed that ViT achieved a lower accuracy of 89.78% and 78.67% for the 5-class and 10-class test sets, respectively. This could be attributed to ViT’s increased focus on global relationships among patches, thereby neglecting the internal features of individual patches. Meanwhile, ViT’s greater demand for sample size also contributed to its lower accuracy.

To explore potential data structures, LSTM with an input data structure different from that of the CNN was selected for the experiment. The LSTM propagates features from the previous to the subsequent time step by using memory cells, thereby facilitating the learning of feature correlations across different time steps. City indicators, upon application to the urban feature data in the experiment, were arranged in a one-dimensional sequential manner for the LSTM. Each sample comprised 1024 time steps, wherein each time step corresponded to a specific feature indicator, thereby resulting in a sample sequence length of 1024. The hidden size was set to 64, and a 2-layer LSTM was utilized. The experimental results indicated that LSTM had a lower accuracy than that of CNN-Custom, with accuracies of 90.89% and 80.67% for the 5-class and 10-class sample test sets, respectively. This can be attributed to LSTM’s inability, particularly for urban features, to adequately explore the correlation between different features across various time steps, especially when the time steps are distant, thereby leading to information decay within memory units. In addition, the sequence order of the urban features was random; therefore, the relative positional relationships between different time steps learned by the LSTM did not have practical significance.

#### Generalization validation

To validate the stability and universality of CNN-Custom in other regional difference studies, similar to urban indicators, features from county-level administration were utilized to assess the economic development levels across counties in China. Official statistics rarely involve county-level administrative areas; therefore, all data used in the experiment were exclusively sourced from the China County Statistical Yearbook for 2019. Similar to the urban feature system, indicators including economic structure, fiscal revenue and expenditure, and financial performance were used as economic indicators, whereas infrastructure, population, area, agricultural output, and industrial scale were used as social indicators. Owing to missing data, the feature system did not contain environmental indicators. Out of the 29 original indicators that were collected, 16 were computed on a per-capita basis, resulting in 45 specific indicators. Following the same data processing approach used for the urban feature data, a 7×7 two-dimensional matrix was constructed using zero-padding. For the sample labels, the regional GDP was chosen as the metric for measuring regional economic competitiveness. Given the large dynamic range of the GDP data, the data range was first compressed via a logarithmic transformation, and then discrete data were obtained via equidistant binning. After removing samples with missing statistics, 2076 original county-level samples were obtained.

Owing to the change in sample size, the convolutional kernel size of the 3rd and 4th convolutional layers of CNN-Custom was changed from four to three, and the stride was changed from two to one to prevent overfitting caused by an excessive abstraction of features. Similarly, the parameters of the DCGAN generator were adjusted based on the sample size. Other methodologies, including standardization, model parameters, dataset splitting ratios, and generated sample ratios, were kept unchanged. In the county-level data test, the test-set accuracy on the 5-class original dataset was 91.46%, which improved to 91.95% through data augmentation (an increase of 0.49%). For the original 10-class dataset, the test set accuracy was 74.88%, which increased to 76.59% through data augmentation, thereby yielding an improvement of 1.71%. Refer to Table 6 for specific experimental results. Overall, the county-level dataset exhibited a favorable performance on the 5-class classification. However, as the number of classifications increased to 10, the accuracy remained relatively low owing to insufficient feature quantity in the samples. The data augmentation method implemented through DCGAN achieved a performance improvement similar to that observed in the urban dataset.

**Table 6:** County-level dataset accuracy.

Dataset	Classification	Original dataset	Augmented dataset	Accuracy improvement
Validation Set	Five-class	89.02%	89.76%	0.74%
	Ten-class	76.59%	76.83%	0.24%
Test Set	Five-class	91.46%	91.95%	0.49%
	Ten-class	74.88%	76.59%	1.71%

#### Data augmentation validation

**Fig 8:**
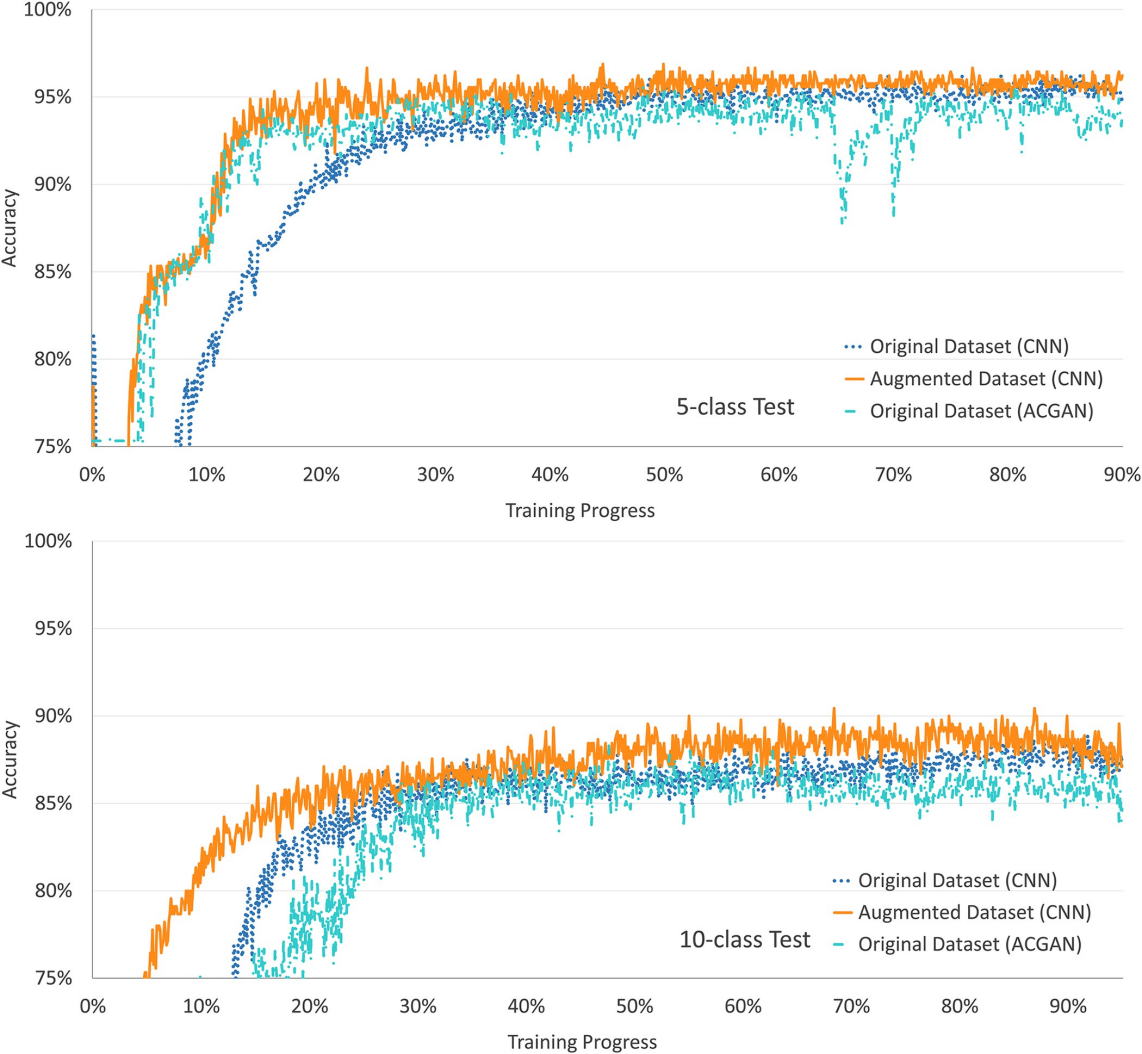
Accuracy comparison of different models and datasets.

To further evaluate the performance of data augmentation methods, the ACGAN was added for comparison purposes. The design idea of the ACGAN was similar to that of this experiment, both of which added generated samples to the classification training so that the classifier could obtain more diverse sample features to increase classification accuracy. The differences are (1) ACGAN is based on CGAN, which can control the category of generated samples, and (2) ACGAN has no ability to filter the generated samples, but directly feeds into the classification training. The experimental results showed that ACGAN attained an accuracy of 94.00% on the 5-class sample test set, slightly lower than the performance on the augmented dataset with CNN-Custom. However, it only achieved an accuracy of 87.56% on the 10-class test set, even lower than the performance of CNN-Custom without data augmentation. The validation accuracy changes of different models and datasets during training, as visually demonstrated in Fig 8, intuitively prove this point. This could be due to the uneven distribution of the original samples, resulting in an ACGAN based on the CGAN generation model, which tends to generate samples of a certain category more often. The uneven distribution of samples was further aggravated by the excessive difference in the quality of the generated samples from different classes. In addition, low-quality generated samples negatively affected the training of the classifier, causing a decrease in the classification accuracy.

Referring to the test results of the experiments for various validations in Tables 6 and 7, it was observed that when dealing with urban feature data, the classification performance of CNN-Custom was comparable to that of VGG and superior to other deep learning models. Its design, which was tailored to the structure of the urban samples, resulted in better generalization and interpretability. However, when applied to other regional difference studies, owing to insufficient regional feature indicators, its performance on tasks with more categories was barely satisfactory. Furthermore, urban feature data contain various potential feature extraction patterns and data structures. However, owing to the randomness in the ordering of indicators, its application in sequence neural networks exhibits a certain level of "black box" nature. Additionally, the inclusion of filtering for the generated samples and the ensemble generation mechanism led to a more stable performance in terms of data augmentation.

**Table 7:** Validate model accuracy.

Dataset	Classification	CNN (custom)	VGG 11	VGG 16	VIT	LSTM	ACGAN
Validation Set	Five-class	96.44%	96.67%	96.22%	92.44%	94.67%	95.56%
	Ten-class	89.11%	89.56%	88.89%	84.89%	82.44%	88.89%
Test Set	Five-class	93.78%	94.89%	94.00%	89.78%	90.89%	94.00%
	Ten-class	88.44%	87.11%	87.11%	78.67%	80.67%	87.56%

## Discussion

This study develops a city economic competitiveness classification model based on deep learning neural networks and complex urban features. It addresses the limitation of traditional economic competitiveness research, which relies on regression models and limited features, and fails to fully explore the interaction effects and nonlinear relationships among features. The study successfully combines a deep learning model based on CNN with complex urban features. The study first constructed a complex city feature system, city feature data were economic, social, and environmental data of 283 prefecture-level cities in China collected between 2012 and 2019. The dataset contained 2264 city samples; each sample comprised 1008 urban characteristic indicators. By dividing the economic competitiveness of cities into five and ten levels, the classification accuracy of the CNN test set reached 93.78% and 88.44%. Additionally, considering the substantial demand for sample size in machine learning, and the fixed number of cities within a region, the study proposes a data augmentation method based on DCGAN. By blending DCGAN-generated samples with original samples, the classification accuracy of CNN was further improved by 0.44% and 2.00%. This study further extends the application of machine learning in urban research, fully exploring the potential of big data-driven urban feature data. It incorporates complex urban features into neural network models, treating cities as complex systems and considering the nonlinear relationships among various systems within cities. In terms of model training, the application of DCGAN enables data augmentation at the sample quantity level, providing a more versatile enhancement approach, especially for research fields that are frequently limited by sample size in regional disparity studies.

## Conclusions

The urban economic competitiveness classification model based on machine learning can provide a more accurate and stable way to identify differences in regional development, thus assisting in making targeted policies and investment decisions. Further expansion of the potential applications in analyzing indicators similar to urban economic competitiveness, such as urban livability and sustainability, might also be achieved through targeted adjustments of feature indicators and experimental samples. In addition, this study has implications for research on other dimensions of regional differences, such as counties and villages. In the future, a deeper integration of economic theoretical frameworks with neural networks and enrichment of data feature engineering will be performed to provide more opportunities for the development of artificial intelligence in urban economic research.

## Supporting information

S1 Appendix Specific indicators and data sources in the complex urban feature system. (PDF)
