# Supplementary material for: Enhancing economic competitiveness analysis through machine learning: Exploring complex urban features
Source: PLoS One. 2023 Nov 7;18(11):e0293303. doi: 10.1371/journal.pone.0293303 (PMC10629647; doi:10.1371/journal.pone.0293303)
Supplement: S1 Appendix — (PDF) [file pone.0293303.s001.pdf]

Specific indicators and data sources in the complex urban feature system (Economy)

Economic Competitiveness Indicators	Item Number	Per Capita Item Number		Basic Economic Performance	Per Capita Calculation	Data Sources
	1		Gross Regional Product (GRP)	Gross Regional Product (Current Prices) (10000 yuan)	N	China City Statistical Yearbook (Total City)
	2			Per Capita GRP (yuan)	N	China City Statistical Yearbook (Total City)
	3			GRP Growth Rate (%)	N	China City Statistical Yearbook (Total City)
	4			Gross Regional Product (Current Prices) (10000 yuan)	N	China City Statistical Yearbook (Districts under City)
	5			Per Capita GRP (yuan)	N	China City Statistical Yearbook (Districts under City)
	6			GRP Growth Rate (%)	N	China City Statistical Yearbook (Districts under City)
	7	A-1	Industrial Enterprise Performance	Number of Industrial Enterprises (unit)	Y	China City Statistical Yearbook (Total City)
	8	A-2		Value-added Tax Payable of Industrial Enterprises	Y	China City Statistical Yearbook (Total City)
	9	A-3		Total Profits of Industrial Enterprises	Y	China City Statistical Yearbook (Total City)
	10	A-4		Total Current Assets of Industrial Enterprises	Y	China City Statistical Yearbook (Total City)
	11	A-5		Gross Industrial Output Value (current price) (10000 yuan)	Y	China City Statistical Yearbook (Total City)
	12	A-6		Total Fixed Assets of Industrial Enterprises (10000 yuan)	Y	China City Statistical Yearbook (Total City)
	13	A-7		Number of Industrial Enterprises (unit)	Y	China City Statistical Yearbook (Districts under City)
	14	A-8		Value-added Tax Payable of Industrial Enterprises (10000 yuan)	Y	China City Statistical Yearbook (Districts under City)
	15	A-9		Total Profits of Industrial Enterprises (10000 yuan)	Y	China City Statistical Yearbook (Districts under City)
	16	A-10		Total Current Assets of Industrial Enterprises (10000 yuan)	Y	China City Statistical Yearbook (Districts under City)
	17	A-11		Total Fixed Assets of Industrial Enterprises (10000 yuan)	Y	China City Statistical Yearbook (Districts under City)
	18	A-12		Tax and Extra Charges from Principal Business of Industrial Enterprises (10000 yuan)	Y	China City Statistical Yearbook (Districts under City)
	19	A-13	Consumer Goods Market Size	Total Retail Sales of Consumer Goods (10000 yuan)	Y	China City Statistical Yearbook (Total City)
	20	A-14		Total Sales of Commodities of Enterprises above Designated Size in Wholesale and Retail Trades (10000 yuan)	Y	China City Statistical Yearbook (Total City)
	21	A-15		Number of Enterprises above Designated Size of Wholesale and Retail Trades (Number of Legal Entities) (unit)	Y	China City Statistical Yearbook (Total City)
	22	A-16		Total Retail Sales of Consumer Goods (10000 yuan)	Y	China City Statistical Yearbook (Districts under City)
	23	A-17		Total Sales of Commodities of Enterprises above Designated Size in Wholesale and Retail Trades (10000 yuan)	Y	China City Statistical Yearbook (Districts under City)
	24	A-18		Number of Enterprises above Designated Size of Wholesale and Retail Trades (Number of Legal Entities) (unit)	Y	China City Statistical Yearbook (Districts under City)

Item Number	Per Capita Item Number		Economic Structure	Per Capita Calculation	Data Sources
25	A-19	Technology Innovation	Number of Scientific and Technological Innovation Personnel	Y	China City Statistical Yearbook (Total City)
26	A-20		Scientific and Technological Innovation Internal Outlay (yuan)	Y	China City Statistical Yearbook (Total City)
27	A-21		Number of Patent Applications (piece)	Y	China City Statistical Yearbook (Total City)
28	A-22		Number of Patent Authorizations (piece)	Y	China City Statistical Yearbook (Total City)
29	A-23		Number of Invention Patent Authorizations (piece)	Y	China City Statistical Yearbook (Total City)
30		GRP Structure	Primary Industry as Percentage to GRP (%)	N	China City Statistical Yearbook (Total City)
31			Secondary Industry as Percentage to GRP (%)	N	China City Statistical Yearbook (Total City)
32			Tertiary Industry as Percentage to GRP (%)	N	China City Statistical Yearbook (Total City)
33			Primary Industry as Percentage to GRP (%)	N	China City Statistical Yearbook (Districts under City)
34			Secondary Industry as Percentage to GRP (%)	N	China City Statistical Yearbook (Districts under City)
35			Tertiary Industry as Percentage to GRP (%)	N	China City Statistical Yearbook (Districts under City)
36	A-24	Sources of Fixed Asset Investment Funds	Fixed Assets Investment in Urban Service Facilities-The Total Funds Actually Available for The Reported Year (100 Million RMB)	Y	China Urban Construction Statistical Yearbook
37	A-25		Fixed Assets Investment in Urban Service Facilities-The Balance of The Previous Year (100 Million RMB)	Y	China Urban Construction Statistical Yearbook
38	A-26		Fixed Assets Investment in Urban Service Facilities-Sources of Fund (100 Million RMB)	Y	China Urban Construction Statistical Yearbook
39	A-27		Fixed Assets Investment in Urban Service Facilities-State Budgetary Fund (100 Million RMB)	Y	China Urban Construction Statistical Yearbook
40	A-28		Fixed Assets Investment in Urban Service Facilities-Central Budgetary Fund (100 Million RMB)	Y	China Urban Construction Statistical Yearbook
41	A-29		Fixed Assets Investment in Urban Service Facilities-Domestic Loan (100 Million RMB)	Y	China Urban Construction Statistical Yearbook
42	A-30		Fixed Assets Investment in Urban Service Facilities-Securities (100 Million RMB)	Y	China Urban Construction Statistical Yearbook
43	A-31		Fixed Assets Investment in Urban Service Facilities-Foreign Investment (100 Million RMB)	Y	China Urban Construction Statistical Yearbook
44	A-32		Fixed Assets Investment in Urban Service Facilities-Foreign Direct Investment (100 Million RMB)	Y	China Urban Construction Statistical Yearbook
45	A-33		Fixed Assets Investment in Urban Service Facilities-Self Raised Funds (100 Million RMB)	Y	China Urban Construction Statistical Yearbook
46	A-34		Fixed Assets Investment in Urban Service Facilities-Self Owned Funds (100 Million RMB)	Y	China Urban Construction Statistical Yearbook
47	A-35		Fixed Assets Investment in Urban Service Facilities-Other Funds (100 Million RMB)	Y	China Urban Construction Statistical Yearbook
48	A-36		Fixed Assets Investment in Urban Service Facilities-Sum Payable This Year (100 Million RMB)	Y	China Urban Construction Statistical Yearbook
49	A-37	Fixed Asset Investment Amount	Fixed Assets Investment in Urban Service Facilities by Industry-Completed Investment of This Year (100 Million RMB)	Y	China Urban Construction Statistical Yearbook
50	A-38		Fixed Assets Investment in Urban Service Facilities by Industry-Completed Investment of This Year-Water Supply (100 Million RMB)	Y	China Urban Construction Statistical Yearbook
51	A-39		Fixed Assets Investment in Urban Service Facilities by Industry-Completed Investment of This Year-Gas Supply (100 Million RMB)	Y	China Urban Construction Statistical Yearbook
52	A-40		Fixed Assets Investment in Urban Service Facilities by Industry-Completed Investment of This Year-Central Heating (100 Million RMB)	Y	China Urban Construction Statistical Yearbook
53	A-41		Fixed Assets Investment in Urban Service Facilities by Industry-Completed Investment of This Year-Rail Transit System (100 Million RMB)	Y	China Urban Construction Statistical Yearbook
54	A-42		Fixed Assets Investment in Urban Service Facilities by Industry-Completed Investment of This YearRoad and Bridge (100 Million RMB)	Y	China Urban Construction Statistical Yearbook
55	A-43		Fixed Assets Investment in Urban Service Facilities by Industry-Completed Investment of This Year-Sewerage (100 Million RMB)	Y	China Urban Construction Statistical Yearbook
56	A-44		Fixed Assets Investment in Urban Service Facilities by Industry-Completed Investment of This Year-Waste-water Treatment and Reuse (100 Million RMB)	Y	China Urban Construction Statistical Yearbook
57	A-45		Fixed Assets Investment in Urban Service Facilities by Industry-Completed Investment of This Year-Sludge Disposal (100 Million RMB)	Y	China Urban Construction Statistical Yearbook
58	A-46		Fixed Assets Investment in Urban Service Facilities by Industry-Completed Investment of This Year-Wastewater Recycled and Reused (100 Million RMB)	Y	China Urban Construction Statistical Yearbook
59	A-47		Fixed Assets Investment in Urban Service Facilities by Industry-Completed Investment of This Year-Landsc-aping (100 Million RMB)	Y	China Urban Construction Statistical Yearbook
60	A-48		Fixed Assets Investment in Urban Service Facilities by Industry-Completed Investment of This Year-Environmental Sanitation (100 Million RMB)	Y	China Urban Construction Statistical Yearbook
61	A-49		Fixed Assets Investment in Urban Service Facilities by Industry-Completed Investment of This Year-Garbage Treatment (100 Million RMB)	Y	China Urban Construction Statistical Yearbook
62	A-50		Fixed Assets Investment in Urban Service Facilities by Industry-Completed Investment of This Year-Other (100 Million RMB)	Y	China Urban Construction Statistical Yearbook
63	A-51		Fixed Assets Investment in Urban Service Facilities-Newly Added Fixed Assetsof This Year (100 Million RMB)	Y	China Urban Construction Statistical Yearbook

Item Number	Per Capita Item Number		Financial Performance	Per Capita Calculation	Data Sources
64	A-52	Deposit and Loan Amount	Loans of National Banking System at Year-end (10000 yuan)	Y	China City Statistical Yearbook (Total City)
65	A-53		Deposits of National Banking System at Year-end (10000 yuan)	Y	China City Statistical Yearbook (Total City)
66	A-54		Household Saving Deposits at Year-end (10000 yuan)	Y	China City Statistical Yearbook (Total City)
67	A-55		Loans of National Banking System at Year-end (10000 yuan)	Y	China City Statistical Yearbook (Districts under City)
68	A-56		Deposits of National Banking System at Year-end (10000 yuan)	Y	China City Statistical Yearbook (Districts under City)
69	A-57		Household Saving Deposits at Year-end (10000 yuan)	Y	China City Statistical Yearbook (Districts under City)
70		Digital Financial	Digital Financial Inclusion Index (Index_aggregate)	N	Institute of Digital Finance, Peking University
71			Digital Financial Inclusion Index (Coverage_breadth)	N	Institute of Digital Finance, Peking University
72			Digital Financial Inclusion Index (Usage_depth)	N	Institute of Digital Finance, Peking University
73			Digital Financial Inclusion Index (Payment)	N	Institute of Digital Finance, Peking University
74			Digital Financial Inclusion Index (Insurance)	N	Institute of Digital Finance, Peking University
75			Digital Financial Inclusion (Indexmonetary_fund)	N	Institute of Digital Finance, Peking University
76			Digital Financial Inclusion Index (investment)	N	Institute of Digital Finance, Peking University
77			Digital Financial Inclusion Index (Credit)	N	Institute of Digital Finance, Peking University
78			Digital Financial Inclusion Index(Credit_investigation)	N	Institute of Digital Finance, Peking University
79			Digital Financial Inclusion Index (Digitization_level)	N	Institute of Digital Finance, Peking University

Item Number	Per Capita Item Number		Marketization and Openness	Per Capita Calculation	Data Sources
80	A-58	Number of Enterprises	Domestic Funded Enterprises (unit)	Y	China City Statistical Yearbook (Total City)
81	A-59		Enterprises with Funds from Hong Kong, Macao and Taiwan (unit)	Y	China City Statistical Yearbook (Total City)
82	A-60		Foreign Funded Enterprises (unit)	Y	China City Statistical Yearbook (Total City)
83	A-61		Domestic Funded Enterprises (unit)	Y	China City Statistical Yearbook (Districts under City)
84	A-62		Enterprises with Funds from Hong Kong, Macao and Taiwan (unit)	Y	China City Statistical Yearbook (Districts under City)
85	A-63		Foreign Funded Enterprises (unit)	Y	China City Statistical Yearbook (Districts under City)
86			Number of Newly Registered Businesses (unit)	N	National Enterprise Credit Information Publicity System
87	A-64	Enterprise Output Value	Domestic Funded Output Value (10000 yuan)	Y	China City Statistical Yearbook (Total City)
88	A-65		Enterprises with Funds from Hong Kong, Macao and Taiwan Output Value (10000 yuan)	Y	China City Statistical Yearbook (Total City)
89	A-66		Foreign Funded Enterprises Output Value (10000 yuan)	Y	China City Statistical Yearbook (Total City)
90	A-67		Number of Projects for Contracted Foreign Direct Investment (unit)	Y	China City Statistical Yearbook (Total City)
91	A-68		Amount of Foreign Capital Actually Utilized (USD 10000)	Y	China City Statistical Yearbook (Total City)
92	A-69		Gross Industrial Output Value (current price) (10000 yuan)	Y	China City Statistical Yearbook (Districts under City)
93	A-70		Domestic Funded Output Value (10000 yuan)	Y	China City Statistical Yearbook (Districts under City)
94	A-71		Enterprises with Funds from Hong Kong, Macao and Taiwan Output Value (10000 yuan)	Y	China City Statistical Yearbook (Districts under City)
95	A-72		Foreign Funded Enterprises Output Value (10000 yuan)	Y	China City Statistical Yearbook (Districts under City)
96	A-73		Number of Projects for Contracted Foreign Direct Investment (unit)	Y	China City Statistical Yearbook (Districts under City)
97	A-74		Amount of Foreign Capital Actually Utilized (USD 10000)	Y	China City Statistical Yearbook (Districts under City)
98		Foreign Trade Amount	Total Import and Export Volume (USD 10000)	N	Provincial Statistical Yearbook
99			Total Import and Export Volume (10000 yuan)	N	Provincial Statistical Yearbook

Item Number	Per Capita Item Number		Land and Real Estate Market.	Per Capita Calculation	Data Sources
100	A-75	Real Estate Market Investment Amount	Investment in Fixed Assets (Excluding Rural Households) (10000 yuan)	Y	China City Statistical Yearbook (Total City)
101	A-76		Investment in Fixed Assets (Excluding Rural Households)	Y	China City Statistical Yearbook (Districts under City)
102	A-77		Investment in Real Estate Development (10000 yuan)	Y	China City Statistical Yearbook (Total City)
103	A-78		Investment in sidential Buildings (10000 yuan)	Y	China City Statistical Yearbook (Total City)
104	A-79		Investment in Real Estate Development (10000 yuan)	Y	China City Statistical Yearbook (Districts under City)
105	A-80		Investment in sidential Buildings (10000 yuan)	Y	China City Statistical Yearbook (Districts under City)
106		Land Market	Area of Land Used for Urban Construction (sq.km.)	N	China City Statistical Yearbook (Districts under City)
107			Land Used for Urban Construction as Percentage to Urban Area (%)	N	China City Statistical Yearbook (Districts under City)
108			Area of Land Used for Living (sq.km.)	N	China City Statistical Yearbook (Districts under City)
109	A-81		Number of Parcel	Y	China Land and Resources Statistical Yearbook
110	A-82		Land Area (hm²)	Y	China Land and Resources Statistical Yearbook
111	A-83		Newly Increased Area (hm²)	Y	China Land and Resources Statistical Yearbook
112	A-84		Number of Plots (parcel)	Y	China Land and Resources Statistical Yearbook
113	A-85		Land Area (hm²)	Y	China Land and Resources Statistical Yearbook
114	A-86		Newly Increased Area (hm²)	Y	China Land and Resources Statistical Yearbook
115	A-87		Number of Plots (parcel)	Y	China Land and Resources Statistical Yearbook
116	A-88		Land Area (hm²)	Y	China Land and Resources Statistical Yearbook
117	A-89		Newly Increased Area (hm²)	Y	China Land and Resources Statistical Yearbook
118	A-90		Transaction Price Value (10000 yuan)	Y	China Land and Resources Statistical Yearbook
119	A-91		Total Amount of Land Supplied (hm²)	Y	China Land and Resources Statistical Yearbook
120	A-92		Land for Industry Mining and Warehousing (hm²)	Y	China Land and Resources Statistical Yearbook
121	A-93		Land for Commercial and Service Uses (hm²)	Y	China Land and Resources Statistical Yearbook
122	A-94		Land for Residential Uses (hm²)	Y	China Land and Resources Statistical Yearbook
123	A-95		Ordinary Commercial House (hm²)	Y	China Land and Resources Statistical Yearbook
124	A-96		Medium and Low Price,Medium and Small Sized Ordinary Commercial Houses (hm²)	Y	China Land and Resources Statistical Yearbook
125	A-97		Economically Affordable House (hm²)	Y	China Land and Resources Statistical Yearbook
126	A-98		Cheap Rent House (hm²)	Y	China Land and Resources Statistical Yearbook
127	A-99		Public Rent House (hm²)	Y	China Land and Resources Statistical Yearbook
128	A-100		High-Grade Residence (hm²)	Y	China Land and Resources Statistical Yearbook
129	A-101		Other Types (hm²)	Y	China Land and Resources Statistical Yearbook
130	A-102		Land for Public Management and Public Services (hm²)	Y	China Land and Resources Statistical Yearbook
131	A-103		Land for Special Uses (hm²)	Y	China Land and Resources Statistical Yearbook
132	A-104		Land for Transport (hm²)	Y	China Land and Resources Statistical Yearbook
133	A-105		Land for Water Conservancy Facilities (hm²)	Y	China Land and Resources Statistical Yearbook
134	A-106		Granting Number of Land (parcel)	Y	China Land and Resources Statistical Yearbook
135	A-107		Granting Area (hm²)	Y	China Land and Resources Statistical Yearbook
136	A-108		Granting Newly Increased (hm²)	Y	China Land and Resources Statistical Yearbook
137	A-109		Granting Transaction Price Value (10000 yuan)	Y	China Land and Resources Statistical Yearbook
138	A-110		Granting through Agreement Number of Land (parcel)	Y	China Land and Resources Statistical Yearbook
139	A-111		Granting through Agreement Area (hm²)	Y	China Land and Resources Statistical Yearbook
140	A-112		Granting through Agreement Newly Increased (hm²)	Y	China Land and Resources Statistical Yearbook
141	A-113		Granting through Agreement Transaction Price Value (10000 yuan)	Y	China Land and Resources Statistical Yearbook
142	A-114		Granting through Bidding Auction and Listing Number of Land Plots/plot (parcel)	Y	China Land and Resources Statistical Yearbook
143	A-115		Granting through Bidding Auction and Listing Area (hm²)	Y	China Land and Resources Statistical Yearbook
144	A-116		Granting through Bidding Auction and Listing Newly Increased (hm²)	Y	China Land and Resources Statistical Yearbook
145	A-117		Granting through Bidding, Auction and Listing Transaction Price Value/104 yuan (10000 yuan)	Y	China Land and Resources Statistical Yearbook
146			Average Land Price (yuan/sq.m.)	N	China Land and Resources Statistical Yearbook
147			Year-on-year Growth Rate of Average Land Price (%)	N	China Land and Resources Statistical Yearbook
148			Land Price of Commercial Land (yuan/sq.m.)	N	China Land and Resources Statistical Yearbook
149			Year-on-year Growth Rate of Commercial Land Price (%)	N	China Land and Resources Statistical Yearbook
150			Land Price of Residential Land (yuan/sq.m.)	N	China Land and Resources Statistical Yearbook
151			Year-on-year Growth Rate of Residential Land Price (%)	N	China Land and Resources Statistical Yearbook
152			Land Price of Industrial Land (yuan/sq.m.)	N	China Land and Resources Statistical Yearbook
153			Year-on-year Growth Rate of Industrial Land Price (%)	N	China Land and Resources Statistical Yearbook
154	A-118		Area of Urban Construction Land (sq.km.)	Y	China Urban Construction Statistical Yearbook
155	A-119		Area of Residential (sq.km.)	Y	China Urban Construction Statistical Yearbook
156	A-120		Area of Administration and Public Services (sq.km.)	Y	China Urban Construction Statistical Yearbook
157	A-121		Area of Commercial and Business Facilities (sq.km.)	Y	China Urban Construction Statistical Yearbook
158	A-122		Area of Industrial, Manufacturing (sq.km.)	Y	China Urban Construction Statistical Yearbook
159	A-123		Area of Logistics and Warehouse (sq.km.)	Y	China Urban Construction Statistical Yearbook
160	A-124		Area of Road, Street and Transportation (sq.km.)	Y	China Urban Construction Statistical Yearbook
161	A-125		Area of Municipal Utilities (sq.km.)	Y	China Urban Construction Statistical Yearbook
162	A-126		Area of Green Space and Square (sq.km.)	Y	China Urban Construction Statistical Yearbook
163	A-127		Area of Land Requisition This Year (sq.km.)	Y	China Urban Construction Statistical Yearbook
164	A-128		Area of Arable Land (sq.km.)	Y	China Urban Construction Statistical Yearbook
165		Average House Price	Average House Price (January) (yuan/sq.m.)	N	China Land and Resources Statistical Yearbook
166			Average House Price (February) (yuan/sq.m.)	N	China Land and Resources Statistical Yearbook
167			Average House Price (March) (yuan/sq.m.)	N	China Land and Resources Statistical Yearbook
168			Average House Price (April) (yuan/sq.m.)	N	China Land and Resources Statistical Yearbook
169			Average House Price (May) (yuan/sq.m.)	N	China Land and Resources Statistical Yearbook
170			Average House Price (June) (yuan/sq.m.)	N	China Land and Resources Statistical Yearbook
171			Average House Price (July) (yuan/sq.m.)	N	China Land and Resources Statistical Yearbook
172			Average House Price (August) (yuan/sq.m.)	N	China Land and Resources Statistical Yearbook
173			Average House Price (September) (yuan/sq.m.)	N	China Land and Resources Statistical Yearbook
174			Average House Price (October) (yuan/sq.m.)	N	China Land and Resources Statistical Yearbook
175			Average House Price (November) (yuan/sq.m.)	N	China Land and Resources Statistical Yearbook
176			Average House Price (December) (yuan/sq.m.)	N	China Land and Resources Statistical Yearbook

Item Number	Per Capita Item Number		Government Finance Structure and Operations	Per Capita Calculation	Data Sources
177	A-129	Financial Revenue and Expenditure Amount	Public Finance Income (10000 yuan)	Y	China City Statistical Yearbook (Total City)
178	A-130		Public Finance Income (10000 yuan)	Y	China City Statistical Yearbook (Districts under City)
179	A-131		Public Finance Expenditure (10000 yuan)	Y	China City Statistical Yearbook (Total City)
180	A-132		Public Finance Expenditure for Science and Technology (10000 yuan)	Y	China City Statistical Yearbook (Total City)
181	A-133		Public Finance Expenditure for Education (10000 yuan)	Y	China City Statistical Yearbook (Total City)
182	A-134		Public Finance Expenditure (10000 yuan)	Y	China City Statistical Yearbook (Districts under City)
183	A-135		Public Finance Expenditure for Science and Technology (10000 yuan)	Y	China City Statistical Yearbook (Districts under City)
184	A-136		Public Finance Expenditure for Education (10000 yuan)	Y	China City Statistical Yearbook (Districts under City)
185	A-137		Urban Construction Investment Debt Issuance (100 Million RMB)	Y	China City Statistical Yearbook (Districts under City)
186	A-138	Number of Public Utility Employees	Persons Employed by Health, Social Security and Social Welfare (10000 persons)	Y	China City Statistical Yearbook (Total City)
187	A-139		Persons Employed by Public Management and Social Organization (10000 persons)	Y	China City Statistical Yearbook (Total City)
188	A-140		Persons Employed by Health, Social Security and Social Welfare (10000 persons)	Y	China City Statistical Yearbook (Districts under City)
189	A-141		Persons Employed by Public Management and Social Organization (10000 persons)	Y	China City Statistical Yearbook (Districts under City)

Item Number	Per Capita Item Number		Resident Income and Employment Distribution	Per Capita Calculation	Data Sources
190		Personnel Employment	Persons Employed in Urban Units at Year-end (person)	N	China City Statistical Yearbook (Total City)
191			Persons Employed in Private Enterprises and Self-Employed Individuals in Urban Areas (person)	N	China City Statistical Yearbook (Total City)
192	A-142		Registered Unemployed Persons in Urban Areas (person)	Y	China City Statistical Yearbook (Total City)
193			Persons Employed in Urban Units at Year-end (person)	N	China City Statistical Yearbook (Districts under City)
194			Persons Employed in Private Enterprises and Self-Employed Individuals in Urban Areas (person)	N	China City Statistical Yearbook (Districts under City)
195	A-143		Registered Unemployed Persons in Urban Areas (person)	Y	China City Statistical Yearbook (Districts under City)
196	A-144		Average Number of Employed Staff and Workers (10000 persons)	Y	China City Statistical Yearbook (Districts under City)
197	A-145		Number of Employees Joining Urban Basic Pension Insurance (10000 persons)	Y	China City Statistical Yearbook (Districts under City)
198	A-146		Number of Employees Joining Urban Basic Medical Care System (10000 persons)	Y	China City Statistical Yearbook (Districts under City)
199	A-147		Persons Covered of Unemployment Insurance (10000 persons)	Y	China City Statistical Yearbook (Districts under City)
200	A-148		Average Number of Employed Staff and Workers (10000 persons)	Y	China City Statistical Yearbook (Total City)
201	A-149		Number of Employees Joining Urban Basic Pension Insurance (10000 persons)	Y	China City Statistical Yearbook (Total City)
202	A-150		Number of Employees Joining Urban Basic Medical Care System (10000 persons)	Y	China City Statistical Yearbook (Total City)
203	A-151		Persons Covered of Unemployment Insurance (10000 persons)	Y	China City Statistical Yearbook (Total City)
204		Employment Structure	Employed Persons by Primary Industry (person)	N	China City Statistical Yearbook (Total City)
205			Employed Persons by Secondary Industry (person)	N	China City Statistical Yearbook (Total City)
206			Employed Persons by Tertiary Industry (person)	N	China City Statistical Yearbook (Total City)
207			Employed Persons by Primary Industry (%)	N	China City Statistical Yearbook (Total City)
208			Employed Persons by Secondary Industry (%)	N	China City Statistical Yearbook (Total City)
209			Employed Persons by Tertiary Industry (%)	N	China City Statistical Yearbook (Total City)
210	A-152		Persons Employed by Primary Industry (10000 persons)	Y	China City Statistical Yearbook (Total City)
211	A-153		Persons Employed by Secondary Industry: Mining (10000 persons)	Y	China City Statistical Yearbook (Total City)
212	A-154		Persons Employed by Manufacturing (10000 persons)	Y	China City Statistical Yearbook (Total City)
213	A-155		Persons Employed by Production and Distribution of Electricity, Gas and Water (10000 persons)	Y	China City Statistical Yearbook (Total City)
214	A-156		Persons Employed by Construction (10000 persons)	Y	China City Statistical Yearbook (Total City)
215	A-157		Persons Employed by Traffic Transport Storage and Post (10000 persons)	Y	China City Statistical Yearbook (Total City)
216	A-158		Persons Employed by Infrmation Transmission Computer Servic and Software (10000 persons)	Y	China City Statistical Yearbook (Total City)
217	A-159		Persons Employed by Wholesale and Retail Trades (10000 persons)	Y	China City Statistical Yearbook (Total City)
218	A-160		Persons Employed by Hotels and Catering Services (10000 persons)	Y	China City Statistical Yearbook (Total City)
219	A-161		Persons Employed by Financial Intermediation (10000 persons)	Y	China City Statistical Yearbook (Total City)
220	A-162		Persons Employed by Real Estate (10000 persons)	Y	China City Statistical Yearbook (Total City)
221	A-163		Persons Employed by Leasing and Business Services (10000 persons)	Y	China City Statistical Yearbook (Total City)
222	A-164		Persons Employed by Scientific Research, Technical Service and Geologic Prospecting (10000	Y	China City Statistical Yearbook (Total City)
223	A-165		Persons Employed by Management of Water Conservancy Environment (10000 persons)	Y	China City Statistical Yearbook (Total City)
224	A-166		Persons Employed by Services to Households and Other Services (10000 persons)	Y	China City Statistical Yearbook (Total City)
225	A-167		Persons Employed by Education (10000 persons)	Y	China City Statistical Yearbook (Total City)
226	A-168		Persons Employed by Culture Sports and Entertainment (10000 persons)	Y	China City Statistical Yearbook (Total City)
227	A-169		Average Number of Employed Staff and Workers (10 000 persons)	Y	China City Statistical Yearbook (Total City)
228			Employed Persons by Primary Industry (person)	N	China City Statistical Yearbook (Districts under City)
229			Employed Persons by Secondary Industry (person)	N	China City Statistical Yearbook (Districts under City)
230			Employed Persons by Tertiary Industry (person)	N	China City Statistical Yearbook (Districts under City)
231			Employed Persons by Primary Industry (%)	N	China City Statistical Yearbook (Districts under City)
232			Employed Persons by Secondary Industry (%)	N	China City Statistical Yearbook (Districts under City)
233			Employed Persons by Tertiary Industry (%)	N	China City Statistical Yearbook (Districts under City)
234	A-170		Persons Employed by Primary Industry (10000 persons)	Y	China City Statistical Yearbook (Districts under City)
235	A-171		Persons Employed by Secondary Industry: Mining (10000 persons)	Y	China City Statistical Yearbook (Districts under City)
236	A-172		Persons Employed by Manufacturing (10000 persons)	Y	China City Statistical Yearbook (Districts under City)
237	A-173		Persons Employed by Production and Distribution of Electricity, Gas and Water (10000 persons)	Y	China City Statistical Yearbook (Districts under City)
238	A-174		Persons Employed by Construction (10000 persons)	Y	China City Statistical Yearbook (Districts under City)
239	A-175		Persons Employed by Traffic,Transport, Storage and Post (10000 persons)	Y	China City Statistical Yearbook (Districts under City)
240	A-176		Persons Employed by Infrmation Transmission,Computer Servic and Software (10000 persons)	Y	China City Statistical Yearbook (Districts under City)
241	A-177		Persons Employed by Wholesale and Retail Trades (10000 persons)	Y	China City Statistical Yearbook (Districts under City)
242	A-178		Persons Employed by Hotels and Catering Services (10000 persons)	Y	China City Statistical Yearbook (Districts under City)
243	A-179		Persons Employed by Financial Intermediation (10000 persons)	Y	China City Statistical Yearbook (Districts under City)
244	A-180		Persons Employed by Real Estatev (10000 persons)	Y	China City Statistical Yearbook (Districts under City)
245	A-181		Persons Employed by Leasing and Business Services (10000 persons)	Y	China City Statistical Yearbook (Districts under City)
246	A-182		Persons Employed by Scientific Research, Technical Service and Geologic Prospecting (10000	Y	China City Statistical Yearbook (Districts under City)
247	A-183		Persons Employed by Management of Water Conservancy, Environment (10000 persons)	Y	China City Statistical Yearbook (Districts under City)
248	A-184		Persons Employed by Services to Households and Other Services (10000 persons)	Y	China City Statistical Yearbook (Districts under City)
249	A-185		Persons Employed by Education (10000 persons)	Y	China City Statistical Yearbook (Districts under City)
250	A-186		Persons Employed by Culture, Sports and Entertainment (10000	Y	China City Statistical Yearbook (Districts under City)
251	A-187		Average Number of Employed Staff and Workers (10000 persons)	Y	China City Statistical Yearbook (Districts under City)
252		Resident Income and Consumption	Total Wage Bill of Employed Staff and Workers (10000 yuan)	N	China City Statistical Yearbook (Total City)
253			Average Wage of Employed Staff and Workers (yuan)	N	China City Statistical Yearbook (Total City)
254			Total Wage Bill of Employed Staff and Workers (10000 yuan)	N	China City Statistical Yearbook (Districts under City)
255			Average Wage of Employed Staff and Workers (yuan)	N	China City Statistical Yearbook (Districts under City)
256			Per Capita Disposable Income (Urban) (yuan/year)	N	China Agriculture Yearbook
257			Per Capita Consumption Expenditure (Urban) (yuan/year)	N	China Agriculture Yearbook
258			Per Capita Disposable Income (Rural) (yuan/year)	N	China Agriculture Yearbook
259			Per Capita Consumption Expenditure (Rural) (yuan/year)	N	China Agriculture Yearbook

Specific indicators and data sources in the complex urban feature system (Society)

Social Competitiveness Indicators	Item Number	Per Capita Item Number		City Size	Per Capita Calculation	Data Sources
	260		Population Size	Population at Year-end (10000 persons)	N	China City Statistical Yearbook (Total City)
	261			Annual Average Population (10000 persons)	N	China City Statistical Yearbook (Total City)
	262			Population Natural Growth Rate (‰)	N	China City Statistical Yearbook (Total City)
	263			Population at Year-end (10 000 persons)	N	China City Statistical Yearbook (Districts under City)
	264			Annual Average Population (10 000 persons)	N	China City Statistical Yearbook (Districts under City)
	265			Population Natural Growth Rate (‰)	N	China City Statistical Yearbook (Districts under City)
	266			Urban Temporary Population (10000 persons)	N	China Urban Construction Statistical Yearbook
	267			Urban Population (10000 persons)	N	China Urban Construction Statistical Yearbook
	268			Urban Temporary Population (10000 persons)	N	China Urban Construction Statistical Yearbook
	269		Urban Built-up Area Size	Population Density (person/sq.km.)	N	China Urban Construction Statistical Yearbook
	270	A-188		Area of Built District (sq.km.)	Y	China Urban Construction Statistical Yearbook
	271			Urban District Population (10000 persons)	N	China Urban Construction Statistical Yearbook
	272			Urban District Area (sq.km.)	N	China Urban Construction Statistical Yearbook
	273			Urban Area (sq.km.)	N	China Urban Construction Statistical Yearbook
	274			Total Land Area of Administrative region (sq.km.)	N	China City Statistical Yearbook (Total City)
	275			Population Density (person/ sq.km.)	N	China City Statistical Yearbook (Total City)
	276			Area of Land Used for Urban Construction (sq.km.)	N	China City Statistical Yearbook (Districts under City)
	277			Area of Built Districts (sq.km.)	N	China City Statistical Yearbook (Districts under City)
	278			Population Density (person/sq.km.)	N	China City Statistical Yearbook (Districts under City)
	279		Nighttime lights Data	Average Nightlight Data Average (January)	N	NPP-VIIRS Nightlight Data
	280			Average Nightlight Data (February)	N	NPP-VIIRS Nightlight Data
	281			Average Nightlight Data (March)	N	NPP-VIIRS Nightlight Data
	282			Average Nightlight Data (April)	N	NPP-VIIRS Nightlight Data
	283			Average Nightlight Data (May)	N	NPP-VIIRS Nightlight Data
	284			Average Nightlight Data (June)	N	NPP-VIIRS Nightlight Data
	285			Average Nightlight Data (July)	N	NPP-VIIRS Nightlight Data
	286			Average Nightlight Data (August)	N	NPP-VIIRS Nightlight Data
	287			Average Nightlight Data (September)	N	NPP-VIIRS Nightlight Data
	288			Average Nightlight Data (October)	N	NPP-VIIRS Nightlight Data
	289			Average Nightlight Data (November)	N	NPP-VIIRS Nightlight Data
	290			Average Nightlight Data (December)	N	NPP-VIIRS Nightlight Data
	291	A-189	Public Cultural Facilities	Number of Public libraries (unit)	Y	China City Statistical Yearbook (Total City)
	292	A-190		Number of theaters and movie theaters (unit)	Y	China City Statistical Yearbook (Total City)
	293	A-191		Total Collections of Public libraries (1000 copies, 1000 pieces)	Y	China City Statistical Yearbook (Total City)
	294	A-192		Collections of Public Libraries per 100 Persons (copy,piece)	Y	China City Statistical Yearbook (Total City)
	295	A-193		Number of Post Offices at Year-end (unit)	Y	China City Statistical Yearbook (Total City)
	296	A-194		Number of theaters and movie theaters (unit)	Y	China City Statistical Yearbook (Districts under City)
	297	A-195		Total Collections of Public libraries (1000 copies, 1000 pieces)	Y	China City Statistical Yearbook (Districts under City)
	298	A-196		Collections of Public Libraries per 100 Persons (copy,piece)	Y	China City Statistical Yearbook (Districts under City)
	299	A-197		Number of museums (unit)	Y	China City Statistical Yearbook (Districts under City)
	300	A-198		Number of stadiums (unit)	Y	China City Statistical Yearbook (Districts under City)
	301	A-199		Number of Post Offices at Year-end (unit)	Y	China City Statistical Yearbook (Districts under City)
	302			Number of Grade A Scenic Spot (unit)	N	Ministry of Culture and Tourism of the People's Republic of China
	303			Scoring of Number of Grade A Scenic Spot (Scoring)	N	Ministry of Culture and Tourism of the People's Republic of China

Item Number	Per Capita Item Number		Urban Construction Scale	Per Capita Calculation	Data Sources
304	A-200	Telecommunications Services	Revenue from Postal Services (10000 yuan)	Y	China City Statistical Yearbook (Total City)
305	A-201		Revenue from Telecommunication Services (10000 yuan)	Y	China City Statistical Yearbook (Total City)
306	A-202		Number of Subscribers of Local Telephones at Year-end (10000 households)	Y	China City Statistical Yearbook (Total City)
307	A-203		Number of Subscribers of Mobile Telephones at Year-end (10 000 households)	Y	China City Statistical Yearbook (Total City)
308	A-204		Number o f Subscribers of Internet Services (10000 households)	Y	China City Statistical Yearbook (Total City)
309	A-205		Number of Subscribers of Local Telephones at Year-end (10000 households)	Y	China City Statistical Yearbook (Districts under City)
310	A-206		Number of Subscribers of Mobile Telephones at Year-end (10000 households)	Y	China City Statistical Yearbook (Districts under City)
311	A-207		Number o f Subscribers of Internet Services (10000 households)	Y	China City Statistical Yearbook (Districts under City)
312	A-208	Water Supply Capacity	Water Supply (10000 tons)	Y	China City Statistical Yearbook (Districts under City)
313	A-209		Water Consumption for Residential Use (10000 tons)	Y	China City Statistical Yearbook (Districts under City)
314			Daily Water Consumption Per Capita (liter)	N	China Urban Construction Statistical Yearbook
315			Water Coverage Rate (%)	N	China Urban Construction Statistical Yearbook
316			Density of Water Supply Pipelines in Built District (kilometer/ sq.km.)	N	China Urban Construction Statistical Yearbook
317			Density of Sewers inmBuilt District (kilometer/sq.km.)	N	China Urban Construction Statistical Yearbook
318	A-210		Urban Water Supply -Integrated Production Capacity (10000m³/day)	Y	China Urban Construction Statistical Yearbook
319	A-211		Urban Water Supply-Underground Water (10000m³/day)	Y	China Urban Construction Statistical Yearbook
320	A-212		Urban Water Supply-Length of Water Supply Pipelines (km)	Y	China Urban Construction Statistical Yearbook
321	A-213		Urban Water Supply-Total Quantity of Water Supply (10000 m³)	Y	China Urban Construction Statistical Yearbook
322	A-214		Urban Water Supply-The Quantity of Water for Production and Operation (10000 m³)	Y	China Urban Construction Statistical Yearbook
323	A-215		Urban Water Supply-The Quantity of Water for Public Service (10000 m³)	Y	China Urban Construction Statistical Yearbook
324	A-216		Urban Water Supply-The Quantity of Water for Household Use (10000 m³)	Y	China Urban Construction Statistical Yearbook
325	A-217		Urban Water Supply-The Quantity of Water for Other Purposes (10000 m³)	Y	China Urban Construction Statistical Yearbook
326	A-218		Urban Water Supply-The Quantitiy of Free Water Supply (10000 m³)	Y	China Urban Construction Statistical Yearbook
327	A-219		Urban Water Supply-Domestic Water Use-Domestic Water Use (10000 m³)	Y	China Urban Construction Statistical Yearbook
328	A-220		Urban Water Supply-The Lossed Water-The Lossed Water (10000 m³)	Y	China Urban Construction Statistical Yearbook
329			Urban Water Supply-Number of Households with Access to Water Supply (unit)	N	China Urban Construction Statistical Yearbook
330			Urban Water Supply-Household User-Household User (unit)	N	China Urban Construction Statistical Yearbook
331			Urban Water Supply-Population with Access to Water Supply (10,000 persons)-Population with Access to Water Supply (10000 persons)	N	China Urban Construction Statistical Yearbook
332	A-221		Public Water Suppliers-Integrated Production Capacity (10000 m³/day)	Y	China Urban Construction Statistical Yearbook
333	A-222		Public Water Suppliers-Underground Water (10000 m³)	Y	China Urban Construction Statistical Yearbook
334	A-223		Public Water Suppliers-Number of Water Plants (unit)	Y	China Urban Construction Statistical Yearbook
335	A-224		Public Water Suppliers-Underground Water (10000m³/day)	Y	China Urban Construction Statistical Yearbook
336	A-225		Public Water Suppliers-Length of Water Supply Pipelines (km)	Y	China Urban Construction Statistical Yearbook
337	A-226		Public Water Suppliers-Total (10000 m³)	Y	China Urban Construction Statistical Yearbook
338	A-227		Public Water Suppliers-Water Sold (10000 m³)	Y	China Urban Construction Statistical Yearbook
339	A-228		Public Water Suppliers-The Quantity of Water for Production and Operation (10000 m³)	Y	China Urban Construction Statistical Yearbook
340	A-229		Public Water Suppliers-The Quantity of Water for Public Service (10000 m³)	Y	China Urban Construction Statistical Yearbook
341	A-230		Public Water Suppliers-The Quantity of Water for Household Use (10000 m³)	Y	China Urban Construction Statistical Yearbook
342	A-231		Public Water Suppliers-The Quantity of Water for Other Purposes (10000 m³)	Y	China Urban Construction Statistical Yearbook
343	A-232		Public Water Suppliers-The Quantitiy of Free Water Supply (10000 m³)	Y	China Urban Construction Statistical Yearbook
344	A-233		Public Water Suppliers-Domestic Water Use (10000 m³)	Y	China Urban Construction Statistical Yearbook
345	A-234		Public Water Suppliers-The Lossed Water (10000 m³)	Y	China Urban Construction Statistical Yearbook
346			Public Water Suppliers-Number of Households with Access to Water Supply (unit)	N	China Urban Construction Statistical Yearbook
347			Public Water Suppliers-Household User (unit)	N	China Urban Construction Statistical Yearbook
348			Public Water Suppliers-Population with Access to Water Supply (10000 persons)	N	China Urban Construction Statistical Yearbook
349	A-235		Suppliers with Self-Built Facilities-Integrated Production Capacity (10000m³/day)	Y	China Urban Construction Statistical Yearbook
350	A-236		Suppliers with Self-Built Facilities-Underground Water(10000m³/day)	Y	China Urban Construction Statistical Yearbook
351	A-237		Suppliers with Self-Built Facilities-Lengthof Water Supply Pipelines (km)	Y	China Urban Construction Statistical Yearbook
352	A-238		Suppliers with Self-Built Facilities Total Quantity of Water Supply (10000m³)	Y	China Urban Construction Statistical Yearbook
353	A-239		Suppliers with Self-Built Facilities-The Quantity of Waterfor Production and Operation (10000m³)	Y	China Urban Construction Statistical Yearbook
354	A-240		Suppliers with Self-Built Facilities-The Quantity of Water for Public Service (10000m³)	Y	China Urban Construction Statistical Yearbook
355	A-241		Suppliers with Self-Built Facilities-The Quantity of Water for Household Use (10000m³)	Y	China Urban Construction Statistical Yearbook
356	A-242		Suppliers with Self-Built Facilities-The Quantity o f Water for Other Purposes (10000m³)	Y	China Urban Construction Statistical Yearbook
357			Suppliers with Self-Built Facilities-Numberof Households with Access to Water Supply (unit)	N	China Urban Construction Statistical Yearbook
358			Suppliers with Self-Built Facilities-Numberof Households with Access to Water Supply-Household User (unit)	N	China Urban Construction Statistical Yearbook
359			Suppliers with Self-Built Facilities-Population with Access tWater Supply (10000 persons)	N	China Urban Construction Statistical Yearbook
360			Urban Water Conservation-Planned Water Consumers (unit)	N	China Urban Construction Statistical Yearbook
361			Urban Water Conservation-Planned Selfproduced Water Consumers (unit)	N	China Urban Construction Statistical Yearbook
362			Urban Water Conservation-Actual Quantity of Water Used-Total (10000m³)	N	China Urban Construction Statistical Yearbook
363			Urban Water Conservation-Actual Quantity of Water Used-Industry (10000m³)	N	China Urban Construction Statistical Yearbook
364			Urban Water Conservation-Actual Quantity of Water Used-Fresh Water Used (10000m³)	N	China Urban Construction Statistical Yearbook
365			Urban Water Conservation-Actual Quantity of Water Used-Fresh Water Used-Industry (10000m³)	N	China Urban Construction Statistical Yearbook
366			Urban Water Conservation-Actual Quantity of Water Used-Water Reused (10000m³)	N	China Urban Construction Statistical Yearbook
367			Urban Water Conservation-Actual Quantity of Water Used-Water Reused -Industry (10000m³)	N	China Urban Construction Statistical Yearbook
368			Urban Water Conservation-Actual Quantity of Water Used-Water Quantity Consumed in Excess of Quota (10000m³)	N	China Urban Construction Statistical Yearbook
369			Urban Water Conservation-Actual Quantity of Water Used-Water Quantity Consumed in Excess of Quota-Reuse Rate (%)	N	China Urban Construction Statistical Yearbook
370			Urban Water Conservation-Actual Quantity of Water Used--Water Quantity Consumed in Excess of Quota-Reuse Rate-Industry (%)	N	China Urban Construction Statistical Yearbook
371			Urban Water Conservation-Water Saved (10000m³)	N	China Urban Construction Statistical Yearbook
372			Urban Water Conservation-Water Saved-Industry (10000m³)	N	China Urban Construction Statistical Yearbook
373			Urban Water Conservation-Total Investment in Water-Saving Measures (10000 yuan)	N	China Urban Construction Statistical Yearbook
374	A-243	Gas Supply Capacity	Urban Natural Gas -Gas Storage Capacity (10,000 m³)	Y	China Urban Construction Statistical Yearbook
375	A-244		Urban Natural Gas -Length of Gas Supply Pipeline (km)	Y	China Urban Construction Statistical Yearbook
376	A-245		Urban Natural Gas -Total Gas Supplied (10000m³)	Y	China Urban Construction Statistical Yearbook
377	A-246		Urban Natural Gas -Quantity Sold (10000m³)	Y	China Urban Construction Statistical Yearbook
378	A-247		Urban Natural Gas -Households (unit)	Y	China Urban Construction Statistical Yearbook
379	A-248		Urban Natural Gas -Quantity Sold -Central Heating (10000m³)	Y	China Urban Construction Statistical Yearbook
380	A-249		Urban Natural Gas -Quantity Sold -Gas Powered Automobiles (10000m³)	Y	China Urban Construction Statistical Yearbook
381	A-250		Urban Natural Gas -Loss Amount (10000m³)	Y	China Urban Construction Statistical Yearbook
382			Urban Natural Gas -Number of Household with Access to Gas (unit)	N	China Urban Construction Statistical Yearbook
383			Urban Natural Gas -Number of Household with Access to Gas -Household User (unit)	N	China Urban Construction Statistical Yearbook
384			Urban Natural Gas -Population with Access to Gas (10000 persons)	N	China Urban Construction Statistical Yearbook
385	A-251		Urban Natural Gas -Gas Stations for CNG Fueled Motor Vehicles (unit)	Y	China Urban Construction Statistical Yearbook
386	A-252		Urban LPG Supply-Gas Storage Capacity (10000 m³)	Y	China Urban Construction Statistical Yearbook
387	A-253		Urban LPG Supply-Length of Gas Supply Pipeline (km)	Y	China Urban Construction Statistical Yearbook
388	A-254		Urban LPG Supply-Total Gas Supplied (Ton)	Y	China Urban Construction Statistical Yearbook
389	A-255		Urban LPG Supply-Quantity Sold (Ton)	Y	China Urban Construction Statistical Yearbook
390	A-256		Urban LPG Supply-Quantity Sold-Households (Ton)	Y	China Urban Construction Statistical Yearbook
391	A-257		Urban LPG Supply-Quantity Sold-Gas Powered Automobiles (Ton)	Y	China Urban Construction Statistical Yearbook
392	A-258		Urban LPG Supply-Loss Amount (Ton)	Y	China Urban Construction Statistical Yearbook
393			Urban LPG Supply-Number of Household with Access to Gas (unit)	N	China Urban Construction Statistical Yearbook
394			Urban LPG Supply Household-Number of Household with Access to Gas-Household User (unit)	N	China Urban Construction Statistical Yearbook
395			Urban LPG Supply-Population with Access to Gas (10,000 persons)	N	China Urban Construction Statistical Yearbook
396	A-259		Urban LPG Supply-Gas Stations for LPG Fueled Motor Vehicles (unit)	Y	China Urban Construction Statistical Yearbook
397			Gas Coverage Rate (%)	N	China Urban Construction Statistical Yearbook
398	A-260		Total Gas Supply (Coal Gas, Natural Gas) (10000 cubic meters)	Y	China City Statistical Yearbook (Districts under City)
399	A-261		Consumption of Gas for Residential Use (10000 cubic meters)	Y	China City Statistical Yearbook (Districts under City)
400			Population with Access To Gas (person)	N	China City Statistical Yearbook (Districts under City)
401	A-262		Liquefied Petroleum Gas Supply (Ton)	Y	China City Statistical Yearbook (Districts under City)
402	A-263		Consumption of Liquefied Petroleum Gas for Residential Use (Ton)	Y	China City Statistical Yearbook (Districts under City)
403			Population with Access To Liquefied Petroleum Gas (person)	N	China City Statistical Yearbook (Districts under City)
404	A-264	Electricity Supply Capacity	Annual Electricity Consumption (10000 kwh)	Y	China City Statistical Yearbook (Districts under City)
405	A-265		Electricity Consumption for Industrial (10000 kwh)	Y	China City Statistical Yearbook (Districts under City)
406	A-266		Household Electricity Consumption for Urban and Rural Residential (10000 kwh)	Y	China City Statistical Yearbook (Districts under City)

Item Number	Per Capita Item Number		Transport Infrastructure	Per Capita Calculation	Data Sources
407	A-267	Passenger Traffic Volume	Total Passenger Traffc	Y	China City Statistical Yearbook (Total City)
408	A-268		Passenger Traffc by Mode of Railway	Y	China City Statistical Yearbook (Total City)
409	A-269		Passenger Traffc by Mode of Highway	Y	China City Statistical Yearbook (Total City)
410	A-270		Passenger Traffc by Mode of waterway	Y	China City Statistical Yearbook (Total City)
411	A-271	Freight Traffic Volume	Passenger Traffc by Mode of Civil Aviation Freight Traffic	Y	China City Statistical Yearbook (Total City)
412	A-272		Freight Traffic (10 000 tons)	Y	China City Statistical Yearbook (Total City)
413	A-273		Railway Freight Traffic (10 000 tons)	Y	China City Statistical Yearbook (Total City)
414	A-274		Highway Freight Traffic (10 000 tons)	Y	China City Statistical Yearbook (Total City)
415	A-275		Waterway Freight Traffic (10 000 tons)	Y	China City Statistical Yearbook (Total City)
416	A-276		Civil Aviation Freight Traffic (Ton)	Y	China City Statistical Yearbook (Total City)
417	A-277	Road Traffic Construction Scale	Area of City Paved Roads at Year-end (10 000 sq.m)	Y	China City Statistical Yearbook (Districts under City)
418			Per Capita Area of Paved Roads in City (sq.m)	N	China City Statistical Yearbook (Districts under City)
419	A-278		Number of Buses and Trolley Buses under Operation at Year-end (unit)	Y	China City Statistical Yearbook (Districts under City)
420	A-279		Total Annual Volume of Passengers Transported by Buses and Trolley Buses (10 000 person-times)	Y	China City Statistical Yearbook (Districts under City)
421	A-280		Number of Taxis at Year-end (unit)	Y	China City Statistical Yearbook (Districts under City)
422	A-281		Number of Public Transportation Vehicles per 10 000 Population (unit)	Y	China City Statistical Yearbook (Districts under City)
423			**Road Surface Area Per Capita (㎡)**	**N**	China Urban Construction Statistical Yearbook
424	A-282		**Urban Road and Bridge-Length of Roads (km)**	Y	China Urban Construction Statistical Yearbook
425	A-283		**Urban Road and Bridge-Surface Area of Roads (10000 ㎡)**	Y	China Urban Construction Statistical Yearbook
426	A-284		**Urban Road and Bridge-Surface Area of Sidewalks (10000 ㎡)**	Y	China Urban Construction Statistical Yearbook
427	A-285		**Urban Road and Bridge-Number of Bridges (unit)**	Y	China Urban Construction Statistical Yearbook
428	A-286		**Urban Road and Bridge-Great Bridge and Grand Bridge (unit)**	Y	China Urban Construction Statistical Yearbook
429	A-287		**Urban Road and Bridge-Intersection (unit)**	Y	China Urban Construction Statistical Yearbook
430	A-288		**Urban Road and Bridge-Number of Road Lamps (unit)**	Y	China Urban Construction Statistical Yearbook
431	A-289		**Urban Road and Bridge-Length of The Road with Street Lamp (km)**	Y	China Urban Construction Statistical Yearbook
432	A-290	Rail Transit Facilities Scale	**Urban Rail Transit System-Length of Lines (km)**	Y	China Urban Construction Statistical Yearbook
433	A-291		**Urban Rail Transit System-Length of Lines -Subway (km)**	Y	China Urban Construction Statistical Yearbook
434	A-292		**Urban Rail Transit System-Length of Lines -Light Rail (km)**	Y	China Urban Construction Statistical Yearbook
435	A-293		**Urban Rail Transit System-Length of Lines -Monorail (km)**	Y	China Urban Construction Statistical Yearbook
436	A-294		**Urban Rail Transit System-Length of Lines-Cable Car (km)**	Y	China Urban Construction Statistical Yearbook
437	A-295		**Urban Rail Transit System-Length of Lines-Maglev (km)**	Y	China Urban Construction Statistical Yearbook
438	A-296		**Urban Rail Transit System-Length of Lines-Surface Lines (km)**	Y	China Urban Construction Statistical Yearbook
439	A-297		**Urban Rail Transit System-Length of Lines-Underground Lines (km)**	Y	China Urban Construction Statistical Yearbook
440	A-298		**Urban Rail Transit System-Length of Lines-Elevated Lines (km)**	Y	China Urban Construction Statistical Yearbook
441	A-299		**Urban Rail Transit System-Number of Stations (unit)**	Y	China Urban Construction Statistical Yearbook
442	A-300		**Urban Rail Transit System-Number of Stations-Surface Lines (unit)**	Y	China Urban Construction Statistical Yearbook
443	A-301		**Urban Rail Transit System-Number of Stations-Underground Lines (unit)**	Y	China Urban Construction Statistical Yearbook
444	A-302		**Urban Rail Transit System-Number of Stations-Elevated Lines (unit)**	Y	China Urban Construction Statistical Yearbook
445	A-303		**Urban Rail Transit System-Number of Transfer Stations (unit)**	Y	China Urban Construction Statistical Yearbook
446	A-304		**Urban Rail Transit System-Number of Vehicles in Service (unit)**	Y	China Urban Construction Statistical Yearbook
447	A-305		**Urban Rail Transit System-Number of Vehicles in Service-Subway (unit)**	Y	China Urban Construction Statistical Yearbook
448	A-306		**Urban Rail Transit System-Number of Vehicles in Service-Light Rail (unit)**	Y	China Urban Construction Statistical Yearbook
449	A-307		**Urban Rail Transit System-Number of Vehicles in Service-Monorail (unit)**	Y	China Urban Construction Statistical Yearbook
450	A-308		**Urban Rail Transit System-Number of Vehicles in Service-Cable Car (unit)**	Y	China Urban Construction Statistical Yearbook
451	A-309		**Urban Rail Transit System-Number of Vehicles in Service-Maglev (unit)**	Y	China Urban Construction Statistical Yearbook
452	A-310		**Urban Rail Transit System (under construction) -Length of Lines (km)**	Y	China Urban Construction Statistical Yearbook
453	A-311		**Urban Rail Transit System (under construction) -Length of Lines -Subway (km)**	Y	China Urban Construction Statistical Yearbook
454	A-312		**Urban Rail Transit System (under construction)-Length of Lines -Light Rail (km)**	Y	China Urban Construction Statistical Yearbook
455	A-313		**Urban Rail Transit System (under construction)-Length of Lines -Monorail (km)**	Y	China Urban Construction Statistical Yearbook
456	A-314		**Urban Rail Transit System (under construction)-Length of Lines -Cable Car (km)**	Y	China Urban Construction Statistical Yearbook
457	A-315		**Urban Rail Transit System (under construction)-Length of Lines -Maglev (km)**	Y	China Urban Construction Statistical Yearbook
458	A-316		**Urban Rail Transit System (under construction)-Length of Lines -Surface Lines (km)**	Y	China Urban Construction Statistical Yearbook
459	A-317		**Urban Rail Transit System (under construction) -Length of Lines -Underground Lines (km)**	Y	China Urban Construction Statistical Yearbook
460	A-318		**Urban Rail Transit System (under construction)-Length of Lines-Elevated (km) Lines**	Y	China Urban Construction Statistical Yearbook
461	A-319		**Urban Rail Transit System (under construction)-Number of Stations-NumberofStations(unit)**	Y	China Urban Construction Statistical Yearbook
462	A-320		**Urban Rail Transit System (under construction)-Number of Stations-Surface Lines(unit)**	Y	China Urban Construction Statistical Yearbook
463	A-321		**Urban Rail Transit System (under construction)-Number of Stations-Underground Lines(unit)**	Y	China Urban Construction Statistical Yearbook
464	A-322		**Urban Rail Transit System (under construction)-Number of Stations-Elevated Lines(unit)**	Y	China Urban Construction Statistical Yearbook
465	A-323		**Urban Rail Transit System (under construction)-Number of Stations-Numberof Transfer Stations (unit)**	Y	China Urban Construction Statistical Yearbook
466	A-324		**Urban Rail Transit System (under construction)-Number of Vehiclesin Service Number of Vehiclesin Service (unit)**	Y	China Urban Construction Statistical Yearbook
467	A-325		**Urban Rail Transit System (under construction)-Number of Vehiclesin Service -Subway (unit)**	Y	China Urban Construction Statistical Yearbook
468	A-326		**Urban Rail Transit System (under construction)-Number ofVehiclesinService -Light Rail (unit)**	Y	China Urban Construction Statistical Yearbook
469	A-327		**Urban Rail Transit System (under construction)-Number of Vehiclesin Service -Monorail (unit)**	Y	China Urban Construction Statistical Yearbook
470	A-328		**Urban Rail Transit System (under construction)-Number of Vehiclesin Service -Cable Car (unit)**	Y	China Urban Construction Statistical Yearbook
471	A-329		**Urban Rail Transit System (under construction)-Number of Vehiclesin Service -Maglev (unit)**	Y	China Urban Construction Statistical Yearbook

Item Number	Per Capita Item Number		Education and Medical Service	Per Capita Calculation	Data Sources
472	A-330	Educational Facilities	Regular Institutions of Higher Education number (unit)	Y	China City Statistical Yearbook (Total City)
473	A-331		Regular Secondary Schools number (unit)	Y	China City Statistical Yearbook (Total City)
474	A-332		Primary Schools number (unit)	Y	China City Statistical Yearbook (Total City)
475	A-333		Vocational Secondary Schools number (unit)	Y	China City Statistical Yearbook (Total City)
476	A-334		Regular Institutions of Higher Education number (unit)	Y	China City Statistical Yearbook (Districts under City)
477	A-335		Regular Secondary Schools number (unit)	Y	China City Statistical Yearbook (Districts under City)
478	A-336		Primary Schools number (unit)	Y	China City Statistical Yearbook (Districts under City)
479	A-337		Vocational Secondary Schools number (unit)	Y	China City Statistical Yearbook (Districts under City)
480	A-338		Number of Full-time Teachers(Regular Institutions of Higher Education) (person)	Y	China City Statistical Yearbook (Districts under City)
481			Number of Higher Education Institutions (Scoring)	N	Educational Statistics Yearbook of China
482			Number of "Project 985" Universities (Scoring)	N	Educational Statistics Yearbook of China
483			Number of "Project 211" Universities (Scoring)	N	Educational Statistics Yearbook of China
484			Number of Project "Double World-Class" Universities (Scoring)	N	Educational Statistics Yearbook of China
485			Overall score of Higher Education Institutions	N	Educational Statistics Yearbook of China
486		Number of Teachers	Number of Full-time Teachers(Regular Secondary Schools) (person)	N	China City Statistical Yearbook (Total City)
487			Number of Full-time Teachers(Regular Secondary (person)	N	China City Statistical Yearbook (Districts under City)
488	A-339		Number of Full-time Teachers(Regular Institutions of Higher Education) (person) (person)	Y	China City Statistical Yearbook (Total City)
489	A-340		Number of Full-time Teachers (Regular Primary Schools) (person)	Y	China City Statistical Yearbook (Total City)
490	A-341		Number of Full-time Teachers (Vocational Secondary Schools) (person)	Y	China City Statistical Yearbook (Total City)
491	A-342		Number of Full-time Teachers(Regular Primary Schools) (person)	Y	China City Statistical Yearbook (Districts under City)
492	A-343		Number of Full-time Teachers(Vocational Secondary Schools) (person)	Y	China City Statistical Yearbook (Districts under City)
493		Number of Students	Number ofStudents Enrollment(Regular Institutions of Higher Education) (10000 persons)	N	China City Statistical Yearbook (Districts under City)
494			Number ofStudents Enrollment (Regular Secondary Schools) (10000 persons)	N	China City Statistical Yearbook (Districts under City)
495			Number ofStudents Enrollment (Regular Primary Schools) (10000 persons)	N	China City Statistical Yearbook (Districts under City)
496			Number of Students in high school stage (person)	N	China City Statistical Yearbook (Districts under City)
497			Number of Students in secondary vocational technical school (person)	N	China City Statistical Yearbook (Districts under City)
498			Number of Students in adult high school (person)	N	China City Statistical Yearbook (Districts under City)
499			Students Enrollment of Regular Institutions of Higher Education Per 10000 persons (person)	N	China City Statistical Yearbook (Districts under City)
500			Students Enrollment of Vocational Secondary Schools Per 10 000 persons (person)	N	China City Statistical Yearbook (Districts under City)
501			Number of Students Enrollment (Regular Institutions of Higher Education) (10000 persons)	N	China City Statistical Yearbook (Total City)
502			Number of Students Enrollment (Regular Secondary Schools) (10000 persons)	N	China City Statistical Yearbook (Total City)
503			Number of Students Enrollment (Regular Primary Schools) (10000 persons)	N	China City Statistical Yearbook (Total City)
504			Number of Students in high school stage (person)	N	China City Statistical Yearbook (Total City)
505			Number of Students in secondary vocational technical school (person)	N	China City Statistical Yearbook (Total City)
506			Number of Students in adult high school (person)	N	China City Statistical Yearbook (Total City)
507			Students Enrollment of Regular Institutions of Higher Education Per 10000 persons (person)	N	China City Statistical Yearbook (Total City)
508			Students Enrollment of Vocational Secondary Schools Per 10000 persons (person)	N	China City Statistical Yearbook (Total City)
509	A-344	Medical Service Providers	Number of Health Care Institution (unit)	Y	China City Statistical Yearbook (Total City)
510	A-345		Number of Hospitals and Health centers (unit)	Y	China City Statistical Yearbook (Total City)
511	A-346		Number of Beds of Hospitals and Health Centers (bed)	Y	China City Statistical Yearbook (Total City)
512	A-347		Number of Doctors (Licensed Doctors + Assistant Doctors) (person)	Y	China City Statistical Yearbook (Total City)
513	A-348		Number of Hospitals and Health centers (unit)	Y	China City Statistical Yearbook (Districts under City)
514	A-349		Number of Beds of Hospitals and Health Centers (bed)	Y	China City Statistical Yearbook (Districts under City)
515	A-350		Number of Doctors (Licensed Doctors + Assistant Doctors) (person)	Y	China City Statistical Yearbook (Districts under City)

Specific indicators and data sources in the complex urban feature system (Environment)

Environmental Competitiveness Indicators	Item Number	Per Capita Item Number		Pollutant Emission Treatment	Per Capita Calculation	Data Sources
	516	A-351	Pollutant Emissions	Volume of Industrial Waste Water Discharged (10000 tons)	Y	China City Statistical Yearbook (Total City)
	517	A-352		Volume of Sulphur Dioxide Emission (ton)	Y	China City Statistical Yearbook (Total City)
	518	A-353		Volume of Industry Sulphur Dioxide Produced (ton)	Y	China City Statistical Yearbook (Total City)
	519	A-354		Urban Drainage and Wastewater Treatment-Quantity of Dry Sludge Produced (ton)	Y	China Urban Construction Statistical Yearbook
	520	A-355		Urban Environmental Sanitation-Domestic Garbage-Quantity of Domestic Garbage (10000 ton)	Y	China Urban Construction Statistical Yearbook
	521	A-356	Pollutant Treatment Volume	Volume of Industrial Soot(dust) remove (ton)	Y	China City Statistical Yearbook (Total City)
	522	A-357		Volume of Industrial Soot(dust) Emission (ton)	Y	China City Statistical Yearbook (Total City)
	523	A-358		Ratio of Consumption Wastes Treated (%)	Y	China City Statistical Yearbook (Total City)
	524	A-359		Ratio of Industrial Solid Wastes Comprehensively Utilized (%)	Y	China City Statistical Yearbook (Total City)
	525	A-360		Ratio of waste Water Centralized Treated of Sewage Work(%)	Y	China City Statistical Yearbook (Total City)
	526			Wastewater Treatment Rate (%)	N	China Urban Construction Statistical Yearbook
	527			Centralized Treatment Rate of Wastewater Treatment Plants (%)	N	China Urban Construction Statistical Yearbook
	528			Domestic Garbage Treatment Rate (%)	N	China Urban Construction Statistical Yearbook
	529			Domestic Garbage Harmless Treatment Rate (%)	N	China Urban Construction Statistical Yearbook
	530	A-361		Urban Drainage and Wastewater Treatment-Annual Quantity of Wastewater Discharged (10000 m^3)	Y	China Urban Construction Statistical Yearbook
	531	A-362		Urban Drainage and Wastewater Treatment-Treatment Capacity (10,000 m^3 /day)	Y	China Urban Construction Statistical Yearbook
	532	A-363		Urban Drainage and Wastewater Treatment-Quantity of Wastewater Treated (10000 m^3)	Y	China Urban Construction Statistical Yearbook
	533	A-364		Urban Drainage and Wastewater Treatment-Secondary and Tertiary Treated (10000 m^3)	Y	China Urban Construction Statistical Yearbook
	534	A-365		Urban Drainage and Wastewater Treatment-Quantity of Dry Sludge Treated (ton)	Y	China Urban Construction Statistical Yearbook
	535	A-366		Urban Drainage and Wastewater Treatment-Other Wastewater Treatment Facilities (10,000 m^3/day)	Y	China Urban Construction Statistical Yearbook
	536	A-367		Urban Drainage and Wastewater Treatment-Quantity Wastewater Treated (10000 m^3)	Y	China Urban Construction Statistical Yearbook
	537	A-368		Urban Drainage and Wastewater Treatment-Total Quantity of Wastewater Treated (10000 m^3)	Y	China Urban Construction Statistical Yearbook
	538	A-369		Urban Drainage and Wastewater Treatment-Recycled Water -Recycled Water Production Capacity (10,000 m^3/day)	Y	China Urban Construction Statistical Yearbook
	539	A-370		Urban Drainage and Wastewater Treatment-Annual Quantity of Wastewater Recycled and Reused (10,000 m^3)	Y	China Urban Construction Statistical Yearbook
	540	A-371		Urban Environmental Sanitation-Volume of Soil Collected and Transported (10,000 ton)	Y	China Urban Construction Statistical Yearbook
	541	A-372		Urban Environmental Sanitation-Quantity of Excrement Treated (10000 ton)	Y	China Urban Construction Statistical Yearbook
	542	A-373		Urban Environmental Sanitation-Domestic Garbage-Harmless Treatment Capacity (ton /day)	Y	China Urban Construction Statistical Yearbook
	543	A-374		Urban Environmental Sanitation-Domestic Garbage-Harmless Treatment Capacity -Sanitary Landfill (ton /day)	Y	China Urban Construction Statistical Yearbook
	544	A-375		Urban Environmental Sanitation-Domestic Garbage-Harmless Treatment Capacity -Incineration (ton /day)	Y	China Urban Construction Statistical Yearbook
	545	A-376		Urban Environmental Sanitation-Domestic Garbage-Harmless Treatment Capacity -other (ton /day)	Y	China Urban Construction Statistical Yearbook
	546	A-377		Urban Environmental Sanitation-Domestic Garbage-Volume of Harmlessly Treated (10000 ton)	Y	China Urban Construction Statistical Yearbook
	547	A-378		Urban Environmental Sanitation-Domestic Garbage - Volume of Harmlessly Treated - Sanitary Landfill (10000 ton)	Y	China Urban Construction Statistical Yearbook
	548	A-379		Urban Environmental Sanitation-Domestic Garbage - Volume of Harmlessly Treated - Incineration (10000 ton)	Y	China Urban Construction Statistical Yearbook
	549	A-380		Urban Environmental Sanitation-Domestic Garbage - Volume of Harmlessly Treated - other (10000 ton)	Y	China Urban Construction Statistical Yearbook
	550	A-381		Urban Environmental Sanitation-Domestic Garbage-Quantity of Garbage Transported by Airtight Vehicle (10000 ton)	Y	China Urban Construction Statistical Yearbook
	551	A-382		Urban Environmental Sanitation-Domestic Garbage-Volume of Treated (10000 ton)	Y	China Urban Construction Statistical Yearbook
	552	A-383		Urban Drainage and Wastewater Treatment-Secondary and Tertiary Treatment	Y	China Urban Construction Statistical Yearbook

Item Number	Per Capita Item Number		Environmental Facilities	Per Capita Calculation	Data Sources
553	A-384	Environmental Protection Facilities	Urban Environmental Sanitation-Domestic Garbage-Numberof Harmless Treatment Plants/ Grounds (unit)	Y	China Urban Construction Statistical Yearbook
554	A-385		Urban Environmental Sanitation-Domestic Garbage-Numberof Harmless Treatment Plants -Sanitary Landfill (unit)	Y	China Urban Construction Statistical Yearbook
555	A-386		Urban Environmental Sanitation-Domestic Garbage-Numberof Harmless Treatment Plants -Incineration (unit)	Y	China Urban Construction Statistical Yearbook
556	A-387		Urban Environmental Sanitation-Domestic Garbage-Numberof Harmless Treatment Plants -other (unit)	Y	China Urban Construction Statistical Yearbook
557	A-388		Urban Drainage and Wastewater Treatment-Length of Piplines (km)	Y	China Urban Construction Statistical Yearbook
558	A-389		Urban Environmental Sanitation-Surface Areaof Roads Cleaned and Maintained (10000 m^2)	Y	China Urban Construction Statistical Yearbook
559	A-390		Urban Environmental Sanitation-Domestic Garbage-Mechanization (10000 ton)	Y	China Urban Construction Statistical Yearbook
560	A-391		Urban Drainage and Wastewater Treatment-Length of Drainage Piplines (km)	Y	China Urban Construction Statistical Yearbook
561	A-392		Urban Drainage and Wastewater Treatment-Sewers (km)	Y	China Urban Construction Statistical Yearbook
562	A-393		Urban Drainage and Wastewater Treatment-Rainwater Drainage Pipeline (km)	Y	China Urban Construction Statistical Yearbook
563	A-394		Urban Drainage and Wastewater Treatment-Combined Drainage Pipeline (km)	Y	China Urban Construction Statistical Yearbook
564	A-395		Urban Drainage and Wastewater Treatment- Wastewater Treatment Plant (unit)	Y	China Urban Construction Statistical Yearbook
565	A-396		Urban Drainage and Wastewater Treatment-Secondary and Tertiary Treatment (unit)	Y	China Urban Construction Statistical Yearbook
566	A-397		Urban Environmental Sanitation-Number of Latrines (unit)	Y	China Urban Construction Statistical Yearbook
567	A-398		Urban Environmental Sanitation-Number of Latrines-Grade Ⅲ and Above (unit)	Y	China Urban Construction Statistical Yearbook
568	A-399		Urban Environmental Sanitation-Numberof Vehiclesand Equipment Designated for Municipal Environmental Sanitation (unit)	Y	China Urban Construction Statistical Yearbook
569	A-400	Urban Greening	Area of Green Land (hm^2)	Y	China City Statistical Yearbook (Districts under City)
570	A-401		Area of Parks and Green Land (hm^2)	Y	China City Statistical Yearbook (Districts under City)
571			Per capita Area of Green Land (hm^2)	N	China City Statistical Yearbook (Districts under City)
572	A-402		Green Covered Area of Completed Area (hm^2)	Y	China City Statistical Yearbook (Districts under City)
573	A-403		Green Covered Area as % of Completed Area (%)	Y	China City Statistical Yearbook (Districts under City)
574			Public Recreational Green Space Per Capita (m^2)	N	China Urban Construction Statistical Yearbook
575			Green Coverage Rate of Built District (%)	N	China Urban Construction Statistical Yearbook
576			Green Space Rate of Built District (%)	N	China Urban Construction Statistical Yearbook
577	A-404		Urban Landscaping -Green Coverage Area (hm^2)	Y	China Urban Construction Statistical Yearbook
578	A-405		Urban Landscaping -Green Coverage Area in Built District (hm^2)	Y	China Urban Construction Statistical Yearbook
579	A-406		Urban Landscaping -Area of Parksand GreenSpace (hm^2)	Y	China Urban Construction Statistical Yearbook
580	A-407		Urban Landscaping -Area of Parksand GreenSpace in Built District (hm^2)	Y	China Urban Construction Statistical Yearbook
581	A-408		Urban Landscaping -Area of Public Recreational GreenSpace (hm^2)	Y	China Urban Construction Statistical Yearbook
582	A-409		Urban Landscaping -Number of Parks (unit)	Y	China Urban Construction Statistical Yearbook
583	A-410		Urban Landscaping -Park Area (hm^2)	Y	China Urban Construction Statistical Yearbook

Item Number	Per Capita Item Number	Environmental Monitorin	Per Capita Calculation	Data Sources
584	Air Quality Monitoring	AQI	N	China National Environmental Monitoring Centre
585		Air Quality Monitoring (CO)	N	China National Environmental Monitoring Centre
586		Air Quality Monitoring (CO_24h)	N	China National Environmental Monitoring Centre
587		Air Quality Monitoring (NO2)	N	China National Environmental Monitoring Centre
588		Air Quality Monitoring (NO2_24h)	N	China National Environmental Monitoring Centre
589		Air Quality Monitoring (O3)	N	China National Environmental Monitoring Centre
590		Air Quality Monitoring (O3_24h)	N	China National Environmental Monitoring Centre
591		Air Quality Monitoring (O3_8h)	N	China National Environmental Monitoring Centre
592		Air Quality Monitoring (O3_8h_24h)	N	China National Environmental Monitoring Centre
593		Air Quality Monitoring (PM10)	N	China National Environmental Monitoring Centre
594		Air Quality Monitoring (PM10_24h)	N	China National Environmental Monitoring Centre
595		Air Quality Monitoring (P㎡.5)	N	China National Environmental Monitoring Centre
596		Air Quality Monitoring (P㎡.5_24h)	N	China National Environmental Monitoring Centre
597		Air Quality Monitoring (SO2)	N	China National Environmental Monitoring Centre
598		Air Quality Monitoring (SO2_24h)	N	China National Environmental Monitoring Centre
